# Association between sleep microarchitecture and cognition in obstructive sleep apnea

**DOI:** 10.1093/sleep/zsae141

**Published:** 2024-06-29

**Authors:** Andrew E Beaudin, Magdy Younes, Bethany Gerardy, Jill K Raneri, A J Marcus Hirsch Allen, Teresa Gomes, Simon Gakwaya, Frédéric Sériès, John Kimoff, Robert P Skomro, Najib T Ayas, Eric E Smith, Patrick J Hanly

**Affiliations:** Department of Clinical Neurosciences, Cumming School of Medicine, University of Calgary, Calgary, AB, Canada; Hotchkiss Brain Institute, Cumming School of Medicine, University of Calgary, Calgary, AB, Canada; Sleep Disorders Center, Misericordia Health Center, University of Manitoba, Winnipeg, Canada; YRT Limited, Winnipeg, Manitoba, Canada; YRT Limited, Winnipeg, Manitoba, Canada; Sleep Centre, Foothills Medical Centre, Calgary AB, Canada; Department of Medicine, Respiratory and Critical Care Divisions, University of British Columbia, Vancouver, BC, Canada; Respiratory Division and Sleep Laboratory, McGill University Health Centre, Montreal, QC, Canada; Unité de recherche en pneumologie, Centre de recherche, Institut Universitaire de Cardiologie et de Pneumologie de Québec, Université Laval, Québec, QC, Canada; Unité de recherche en pneumologie, Centre de recherche, Institut Universitaire de Cardiologie et de Pneumologie de Québec, Université Laval, Québec, QC, Canada; Respiratory Division and Sleep Laboratory, McGill University Health Centre, Montreal, QC, Canada; Division of Respirology, Critical Care and Sleep Medicine, University of Saskatchewan, Saskatoon, SK, Canada; Department of Medicine, Respiratory and Critical Care Divisions, University of British Columbia, Vancouver, BC, Canada; Department of Clinical Neurosciences, Cumming School of Medicine, University of Calgary, Calgary, AB, Canada; Hotchkiss Brain Institute, Cumming School of Medicine, University of Calgary, Calgary, AB, Canada; Hotchkiss Brain Institute, Cumming School of Medicine, University of Calgary, Calgary, AB, Canada; Sleep Centre, Foothills Medical Centre, Calgary AB, Canada; Department of Medicine, Cumming School of Medicine, University of Calgary, Calgary, AB, Canada

**Keywords:** obstructive sleep apnea, cognition, sleep microarchitecture, spindles, odds-ratio product, mediation

## Abstract

**Study Objectives:**

Obstructive sleep apnea (OSA) increases the risk of cognitive impairment. Measures of sleep microarchitecture from EEG may help identify patients at risk of this complication.

**Methods:**

Participants with suspected OSA (*n* = 1142) underwent in-laboratory polysomnography and completed sleep and medical history questionnaires, and tests of global cognition (Montreal Cognitive Assessment, MoCA), memory (Rey Auditory Verbal Learning Test, RAVLT) and information processing speed (Digit–Symbol Coding, DSC). Associations between cognitive scores and stage 2 non-rapid eye movement (NREM) sleep spindle density, power, frequency and %-fast (12–16Hz), odds-ratio product (ORP), normalized EEG power (EEG_NP_), and the delta:alpha ratio were assessed using multivariable linear regression (MLR) adjusted for age, sex, education, and total sleep time. Mediation analyses were performed to determine if sleep microarchitecture indices mediate the negative effect of OSA on cognition.

**Results:**

All spindle characteristics were lower in participants with moderate and severe OSA (*p* ≤ .001, vs. no/mild OSA) and positively associated with MoCA, RAVLT, and DSC scores (false discovery rate corrected *p*-value, *q* ≤ 0.026), except spindle power which was not associated with RAVLT (*q* = 0.185). ORP during NREM sleep (ORP_NREM_) was highest in severe OSA participants (*p* ≤ .001) but neither ORP_NREM_ (*q* ≥ 0.230) nor the delta:alpha ratio were associated with cognitive scores in MLR analyses (*q* ≥ 0.166). In mediation analyses, spindle density and EEG_NP_ (*p* ≥ .048) mediated moderate-to-severe OSA’s negative effect on MoCA scores while ORP_NREM_, spindle power, and %-fast spindles mediated OSA’s negative effect on DSC scores (*p* ≤ .018).

**Conclusions:**

Altered spindle activity, ORP and normalized EEG power may be important contributors to cognitive deficits in patients with OSA.

## Graphical Abstract

**Graphical Abstract:**
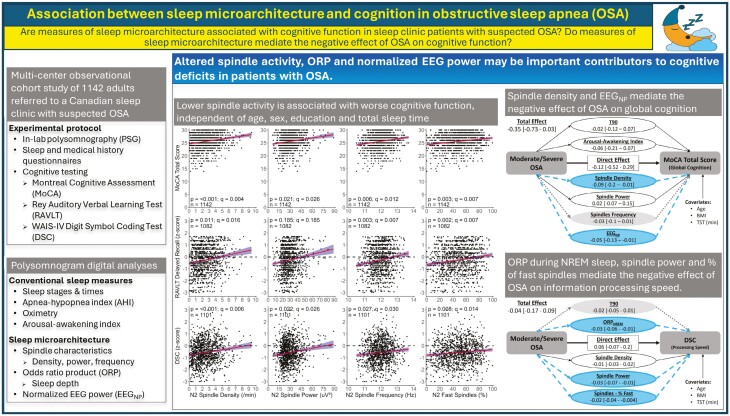

**Statement of Significance**

Obstructive sleep apnea (OSA) increases the risk of cognitive impairment and dementia, but neither conventional measures of OSA severity nor symptom subtypes adequately identify patients with cognitive deficits and additional biomarkers are needed. Using data from a large cohort of sleep clinic patients (*n* = 1142) we investigated whether measures of sleep microarchitecture (spindles, odds-ratio product ([ORP], and normalized EEG power [EEG_NP_]) may provide these additional biomarkers. We found that spindle density and EEG_NP_ mediated OSA’s negative effect on global cognition while spindle power and frequency, and ORP during NREM sleep mediated OSA’s negative effect on information processing speed, suggesting OSA-related differences in sleep microarchitecture, particularly spindle activity, are potential biomarkers to identify patients at the greatest risk of cognitive impairment.

Obstructive sleep apnea (OSA) is a common sleep disorder with ~38% of the world’s population 30–69 years of age having OSA (apnea–hypopnea index (AHI) > 5 events/hour) and ~18% having the moderate-to-severe disease (AHI > 15 events/hour) [1]. OSA is an independent risk factor for cardiovascular, cerebrovascular, metabolic, and kidney disease [2–5], and contributes to the development, and progression, of neurocognitive decline [6]. Deficits in memory, executive function, attention, vigilance, and visuospatial cognitive functions are commonly reported in patients with OSA [7–9]. Moreover, cognitive impairment is highly prevalent in sleep clinic patients with moderate and severe OSA [10], and although untreated OSA predisposes individuals to cognitive decline, not all patients develop this sequelae, the pathogenesis of which is not fully understood [8, 11].

Cognitive impairment in OSA patients is traditionally attributed to chronic nocturnal hypoxemia and sleep fragmentation [12, 13]. Although these exposures likely contribute to the pathogenesis of cognitive deficits, the relationships between cognitive deficits and conventional metrics of OSA severity such as the AHI, indices of nocturnal hypoxemia, and arousals from sleep are modest at best [10, 13–16]. Furthermore, OSA symptom subtypes (e.g. minimally symptomatic, excessively sleepy, disturbed sleep, and excessively sleep with disturbed sleep) do not distinguish between patients with, and without, cognitive deficits [17]. Consequently, additional biomarkers are needed to better identify OSA patients at greatest risk of cognitive impairment.

Advanced quantitative electroencephalography (qEEG) measures of sleep microarchitecture associated with reduced cognitive performance and age-related cognitive decline and neurodegeneration [18–22] may provide these additional markers. Among the numerous potential qEEG that have been described [19], patients with OSA have lower spindle density and power, slower spindle frequency, and reduced overall EEG power [22–24]. Notably, reduced spindle density and EEG power are associated with impaired vigilance and driving performance in patients with OSA [25]. Two additional novel qEEG measures of neuronal activity associated with cognitive impairment and altered in patients with OSA include normalized overall EEG power and the delta:alpha ratio. Normalized EEG power (EEG_NP_) is the average of all power ratios for frequency bands relevant to sleep–wake states (i.e. delta, theta, alpha, sigma, beta, and gamma), where the power ratio for each frequency band is calculated as the quotient between the absolute power within a band and the mean power for that band within the entire study cohort [26]; it is reduced in patients with OSA [26] and a predictor of cognitive decline in older women [22]. The delta:alpha ratio (D/A ratio) is the quotient between absolute delta and alpha powers and is elevated in patients with OSA [27, 28]. Moreover, similar to spindle characteristics and EEG power, an elevated D/A ratio is associated with impaired vigilance [29]. However, associations between qEEG metrics with measures of global cognitive function, memory, or information processing speed in an adult sleep clinic population have not been investigated.

In addition to qEEG measures, the odds-ratio product (ORP) is a novel, validated, continuous measure of sleep depth [30]. In contrast to conventional measures of sleep depth that are expressed as the time spent in non-rapid eye movement (NREM) stages 1–3 and REM sleep [31], the ORP provides a more detailed and specific assessment of sleep depth that is expressed numerically with a range from 0 (very deep sleep) to 2.5 (full wakefulness). To the extent that sleep depth impacts cognitive function [32], the ORP may help identify OSA patients at risk of cognitive impairment.

Using data from a large, well phenotyped, cohort of sleep clinic patients with suspected OSA [10], the objectives of this study were to investigate the associations between cognitive function and qEEG measures of sleep microarchitecture, including the ORP, and to determine whether any of these indices mediate the relationship between OSA and cognitive impairment [10].

## Methods

This study included adults referred to one of the five Canadian sleep centers for suspected OSA between July 2016 and June 2021 or who had a known OSA diagnosis without treatment for at least the previous 6 months who were enrolled into the Canadian Sleep and Circadian Network’s (CSCN) adult OSA observational cohort. OSA was diagnosed by either unattended home sleep apnea testing or in-laboratory polysomnography (PSG; full-night, or split-night) and participants completed a comprehensive sleep questionnaire and underwent cognitive testing prior to initiation of treatment for OSA. This study was approved by the University of British Columbia Clinical Research Ethics Board (H16-00422), Conjoint Health Research Ethics Board of the University of Calgary (REB16-0211), Biomedical Research Ethics Board of the University of Saskatchewan (BIO-REB16-106), McGill University Health Center (MEO-10-2019-4718), and Institut Universataire de Cardiologie et de Pneumologie de Quebec at Université Laval (MP-10-2018-2938). All participants were informed of study requirements and provided written informed consent.

The current study included only participants who underwent full-night or split-night PSG. Polysomnography, sleep questionnaire, and cognitive testing details have been previously described [2, 10, 17, 33] and are summarized below.

### Polysomnography

Polysomnography was performed according to American Academy of Sleep Medicine (AASM) guidelines for clinical diagnosis of OSA (Sandman, Tyco Healthcare, Kanata, ON, Canada; Sleepware G3, Philips Healthcare, Amsterdam, Netherlands; or Polysmith, Nihon Kohden, Irvine, CA, USA) [31]. Recordings included electroencephalography (EEG) channels (C3, C4, M1, M2, O1, and O2), left and right electro-oculograms, submental and bilateral tibialis anterior electromyograms (EMG; surface electrodes), airflow using nasal pressure and oral thermistor, chest and abdomen respiratory efforts using inductance plethysmography and SpO_2_. All channels were continuously recorded at frequencies recommended by the AASM and stored electronically for subsequent manual scoring by registered polysomnographic technologists (RPSGT) according to AASM criteria [31].

### Sleep questionnaire

The sleep questionnaire included demographics (age, sex, height, and weight), medical history, comorbidities, medications, and sleep schedule questions. Insomnia and restless legs syndrome (RLS) were determined using the Insomnia Severity Index (ISI) [34] and the International Restless Legs Scale [35]. The questionnaire also included the Epworth Sleepiness Scale (ESS) [36] and the Pittsburgh Sleep Quality Index (PSQI) [37] to assess daytime sleepiness and sleep quality, respectively. Additional details are provided in Supplementary Material.

### Cognitive testing

The cognitive test battery assessed global cognitive function with the Montreal Cognitive Assessment (MoCA) [38], episodic memory using the Rey Auditory Verbal Learning Test [39] (RAVLT), and information processing speed with the Wechsler Adult Intelligence Scale-4th Edition Digit–Symbol Coding (DSC) subtest [40]. All tests were done in person with administration and scoring standardized across all five centers. Details of individual tests are provided in Supplementary Material.

### Polysomnogram digital analyses

Diagnostic full-night and split-night PSG records were de-identified, converted to European Data Format, and sent to a co-investigator (MY) who was blinded to cognitive testing results, for automated digital analyses. Analyses included conventional scoring metrics, spindle detection, and qEEG analyses, including ORP. Full-night PSGs were divided into two halves with analyses performed on the first half of the study to align with the analysis of split-night PSGs which was performed only on the diagnostic portion (i.e. first half of PSG record).

#### Conventional sleep metrics.

Sleep times, sleep efficiency, sleep stages, AHI, oximetry, and the arousal-awakening index were derived using the validated, semi-automated Michele Sleep Scoring (MSS) software (YRT Limited, Winnipeg, MB, Canada) [41, 42] following AASM guidelines [31]. These results were manually edited by a RPSGT for problematic 30-second epochs identified by the software [42]. An obstructive apnea was defined as a decrease in respiratory airflow of ≥90% for ≥10 seconds with continued (or increasing) respiratory efforts, and an obstructive hypopnea was defined as a decrease in respiratory airflow of ≥30% lasting for ≥10 seconds followed by a decrease in SpO_2_ of ≥3% or an arousal from sleep. Prior to automated scoring the MSS software down samples all EEG channels to 120 Hz, and applies a standard 0.33 Hz high-pass filter and 35.0 Hz low-pass filter to all EEG channels.

#### Sleep spindle detection.

Spindles and their characteristics (density, power, and frequency) were detected from artifact-free central EEG signals (C3 and C4) by a validated algorithm in the MSS software [18, 43]. For each electrode, the algorithm identifies spindles by applying a fast Fourier transform (FFT) to 1-second windows with the FFT window advancing 0.2 seconds at a time such that five spectrograms are derived for each second of data. The total power across the spindle frequency range is then calculated for each 1-second epoch (termed power S). The spindle frequency range is set at 10–16 Hz, except when the average power within the 7.3–12 Hz range (i.e. alpha) in NREM is >15 uV^2^, which is interpreted as alpha intrusion. In this case, spindle frequency range is set to 12–16 Hz. Next, the power S within each of the 150 1-second epochs within a 30-second period are divided by the 30th percentile of power S calculated for each 30-second period (termed power S ratio). A spindle is presumed detected if the power S ratio is >3 for five consecutive 1-second epochs provided the ratio drops below 1.5 or power S decreases to <20% of peak power S (whichever is higher) within 5 seconds [43]. These thresholds were derived using an iterative trial-and-error process that achieved optimal agreement between digital and manual spindle detection within a set of PSGs independent of those used in the current study [18]. Presumed spindles are then excluded if they occur during an arousal or if the power S ratio for the presumptive spindle is less than the ratios for alpha and beta powers and their respective reference values (i.e. 30th percentile of the 30-second epoch).

Spindle density for the C3 and C4 electrodes during NREM stage 2 (N2) sleep was calculated as the total number of detected spindles divided by the time (in minutes) of N2 sleep. For each spindle, spindle power was defined as the highest power S and spindle frequency was defined as the EEG frequency with the highest power. Power and frequency were averaged across all validated spindles detected for each electrode. Fast spindles were defined as those with a frequency 12–16Hz. The limit of > 12Hz is lower than the conventional threshold of > 13Hz because the frequency with the highest power is slower than the highest frequency within the spindle. The percentage of fast spindles for each electrode was calculated by dividing the number of fast spindles by the total number of spindles detected, multiplied by 100 (%-fast).

#### Odds-ratio product.

Odds-ratio product (ORP) was calculated from artifact-free 3-second non-overlapping EEG epochs. ORP is the probability of the spectral pattern in a 3-second epoch being found in 30-second epochs staged wake, or during an arousal as determined by experienced RPSGTs [30, 44] and ranges from 0 (very deep sleep) to 2.5 (full wakefulness) [30]. Briefly, each 3-second EEG epoch undergoes a FFT analysis to generate power in the slow delta band (0.33–2.33 Hz), fast delta + theta band (2.67–6.33 Hz), alpha-sigma band (7.33–14.0 Hz), and in the beta band (14.33 to 35.0 Hz). The power in each of these four frequency bands is assigned a rank (range: 0–9) based upon its location within a reference power range for the respective bands derived from >400 000 artifact-free 3-second epochs collected from clinical PSGs. Next, the four ranks in each 3-second epoch are concatenated to create a four-digit “bin” number. The probability of each of the 10 000 bin numbers occurring in wake epochs or inside an arousal is determined from a reference table. The reference table was generated from manual scoring of the same PSGs used to generate the 10 000 bin numbers by two experienced RPSGTs. The probability (in percent) is divided by 40 (% time in stage wake in the development files) to produce ORP in the 3-second epoch. The 10 ORP values within a 30-second epoch are then averaged to provide an ORP value for each 30-second epoch. Additional details related to the derivation of the ORP are outlined in prior publications [30, 44]. Using the conventional sleep staging from the MSS software a mean ORP is calculated for total recording time (ORP_TRT_), and during wakefulness (ORP_WAKE_) and NREM sleep (ORP_NREM_).

#### Quantitative EEG analyses.

Quantitative EEG measures were determined for both C3 and C4 electrodes from the power spectral analyses performed on the same artifact-free 3-second EEG segments used to derive the ORP. The absolute powers within the slow delta (0.33–2.33 Hz), fast delta + theta (2.67–6.33 Hz), alpha (7.33–12.0 Hz), sigma (12.3–14.0 Hz), beta-1 (14.3–20.0 Hz), beta-2 (20.3–35.0 Hz), and gamma (35.3–60.0 Hz) frequency bands were calculated in each 3-second interval and the values in each band over the entire PSG were averaged after excluding the highest 10% of values, which typically contain artifacts. Relative power for each frequency band was calculated by dividing the absolute power within each band by the total summed power across all bands and multiplying by 100. Normalized power within each of the seven frequency bands was calculated by dividing the absolute power of each band by the cohort average power of that band. Next, all seven ratios were averaged to derive a measure of overall normalized EEG power (EEG_NP_) [26]. Finally, the delta/alpha ratio (D/A ratio) was calculated as the quotient of absolute slow delta and alpha powers [45]. The slow delta (0.33–2.33 Hz) and fast delta + theta (2.67–6.33 Hz) bands were examined rather than the more commonly used delta (0.5–4 Hz) and theta (4–8 Hz) bands because during development of the ORP it was found that power in the slow delta frequency (<2.33 Hz) had little association with the likelihood of a 3-second epoch being analyzed occurring in stage wake, while the combination of fast delta and theta had a strong correlation [30]. In addition, the slow delta and fast delta + theta bands are critical for determining sleep depth to distinguish between slow delta frequencies unrelated to sleep depth from fast frequencies that are very sensitive to sleep depth [46].

### Data analyses

An ISI ≥ 15 indicated the presence of insomnia [34] and a participant was categorized as having RLS if they reported having all of the following symptoms [47]: an urge to move their legs due to uncomfortable or unpleasant sensations in their legs that begin or worsen during periods of rest or sleep and are improved by movement, and that are worse in the evening or night, or only occur during the evening and night. An ESS > 10 indicated excessive daytime sleepiness [36] and a PSQI > 5 indicated poor sleep quality [37]. Sleep duration was quantified from the PSQI question 4 (“How many hours of actual sleep do you get at night?”) with ≤6 hours of sleep per night considered to be short sleep duration [48].

Similar to our prior study [10], a MoCA total score <26 (range: 0–30) was operationally defined to indicate the presence of MCI [38]. Raw RAVLT delayed free recall and DSC scores were converted to z-scores using age-matched normative values [49–51]. Self-reported medications were categorized according to their drug classification [52]. For clinical relevance, we report only medications known to impact cognition [53] (Table 1).

**Table 1.** Participant Characteristics for Entire Cohort and According to OSA Severity

	Entire cohort	No/Mild OSA	Moderate OSA	Severe OSA
*N*	1142	443	257	442
Female, *n* (%)	539 (47.2)	250 (56.4)	119 (46.3)^*^	170 (38.5)^*^
Age (y)	54.0 (44.0–63.0)	51 (41.5–60.5)	55 (45.0–64.0)^*^	56.0 (46.0–65.0)^*^
BMI (kg/m²)	33.7 ± 8.4	30.5 ± 7.1	32.5 ± 6.7^*^	37.6 ± 9.0^*†^
White, *n* (%)	906 (79.3)	343 (77.4)	204 (79.4)	359 (81.2)
*OSA severity*				
AHI (events/h)	21.5 (10.0–47.0)	8.4 (5.2–11.2)	21.5^*^ (18.3–25.2)	56.0^*†^ (41.3–86.2)
Mean SpO_2_ (%)	92.7 (90.3–94.6)	94.1 (92.4–95.5)	92.8^*^ (91.0–94.7)	90.5^*†^ (87.6–92.9)
T90 (% TST)	8.6 (0.5–63.1)	0.6 (0.0–8.1)	6.9^*^ (1.1–39.6)	57.1^*†^ (10.9–83.9)
Minimum SpO_2_ (%)	83.0 (76.0–87.0)	86.0 (83.0–89.0)	83.0^*^ (78.0–87.0)	77.0^*†^ (70.0–82.0)
*Daytime sleepiness and sleep quality*				
ESS score	9.3 ± 5.0	8.9 ± 4.9	8.7 ± 4.7	10.1 ± 5.2^*†^
*ESS > 10, n (%)*	515 (47.2)	174 (41.6)	103 (42.6)	238 (55.1)^*†^
PSQI global score	8.9 ± 3.8	8.7 ± 3.8	8.8 ± 3.6	9.1 ± 3.9
*PSQ I > 5, n (%)*	923 (86.3)	346 (84.2)	211 (89.0)	366 (86.7)
Sleep duration (h)	6.5 ± 1.5	6.5 ± 1.2	6.4 ± 1.4	6.4 ± 1.7
*Sleep ≤ 6 hours, n (%)*	503 (45.2)	170 (39.7)	115 (46.4)	218 (50.0)^*^
ISI total score	12.6 ± 5.7	12.3 ± 5.4	12.3 ± 5.6	13.0 ± 6.1
*ISI > 15, n (%)*	407 (37.7)	144 (34.8)	84 (35.1)	179 (41.9)
RLS (%)	268 (24.4)	100 (23.6)	56 (23.0)	112 (26.0)
*RLS severity, n (%)*	4.7 ± 1.6	4.5 ± 1.5	4.9 ± 1.7	4.9 ± 1.6
*Comorbidities*				
Smoking				
*Never smoker, n (%)*	585 (52.2)	258 (59.6)	120 (48.0)^*^	207 (47.4)^*^
*Past smoker, n (%)*	409 (36.5)	127 (29.3)	100 (40.0)^*^	182 (41.6)^*^
*Current smoker, n (%)*	126 (11.2)	48 (11.1)	30 (12.0)	48 (11.0)
Hypertension, *n* (%)	472 (42.3)	128 (29.7)	113 (45.4)^*^	231 (52.9)^*^
High cholesterol, *n* (%)	393 (35.3)	108 (25.2)	100 (40.0)^*^	185 (42.4)^*^
Diabetes, *n* (%)	199 (17.8)	56 (13.0)	45 (18.1)	98 (22.5)^*^
Kidney disease, *n* (%)	95 (8.5)	37 (8.5)	20 (8.0)	38 (8.7)
Coronary artery disease, *n* (%)	96 (8.6)	20 (4.7)	25 (10.0)^*^	51 (11.7)^*^
Heart failure, *n* (%)	45 (4.0)	11 (2.6)	12 (4.8)	22 (5.0)
Atrial fibrillation, *n* (%)	69 (6.2)	26 (6.1)	14 (5.7)	29 (6.7)
Prior stroke, *n* (%)	36 (3.2)	11 (2.6)	7 (2.8)	18 (4.1)
COPD, *n* (%)	69 (6.2)	22 (5.1)	14 (5.6)	33 (7.6)
Asthma, *n* (%)	234 (21.0)	96 (22.4)	42 (16.8)	96 (22.2)
*Medications*				
Statins, *n* (%)	277 (25.0)	66 (15.4)	70 (28.5)^*^	141 (32.5)^*^
Antidepressants, *n* (%)	243 (21.9)	93 (21.7)	56 (22.8)	94 (21.7)
Gabapentinoid, *n* (%)	64 (5.7)	18 (4.2)	14 (5.6)	32 (7.3)
Opioids, *n* (%)	56 (5.1)	19 (4.4)	9 (3.7)	28 (6.5)
Non-benzodiazepines, *n* (%)	47 (4.2)	23 (5.4)	10 (4.1)	14 (3.2)
Atypical Antipsychotic, *n* (%)	46 (4.2)	15 (3.5)	10 (4.1)	21 (4.8)
Benzodiazepines, *n* (%)	32 (2.9)	11 (2.6)	7 (2.8)	14 (3.2)
Cannabinoids, *n* (%)	20 (1.8)	10 (2.3)	1 (0.4)	9 (2.1)
Dopaminergic, *n* (%)	20 (1.8)	5 (1.2)	8 (3.3)	7 (1.6)

Mean ± SD or median (interquartile range); ^*^p ≤ .05 versus no/mild OSA; ^†^*p* ≤ .05 versus moderate OSA.

*Abbreviations:* BMI, body mass index; AHI, apnea-hypopnea index; Mean SpO_2_ mean arterial oxyhemoglobin saturation across the total sleep time; T90, percentage of total sleep time with SpO_2_ < 90%; ESS, Epworth Sleepiness Scale; PSQI, Pittsburgh Sleep Quality Index; ISI, insomnia severity index; RLS, restless leg syndrome; COPD, chronic obstructive pulmonary disease.

Sleep times, sleep efficiency, sleep stages, AHI, and oximetry derived from the MSS software were used in all analyses. Participants were categorized as having no/mild (AHI < 15 events/hour), moderate (15 ≥ AHI ≤ 30 events/hour), and severe OSA (AHI > 30 events/hour). No and mild OSA participants were pooled because in our previous study of 1084 CSCN participants (702 included in the current study), mild OSA was not associated with higher odds of cognitive impairment (MoCA < 26) compared to participants with no OSA [10]. Mean SpO_2_ during sleep, the percentage of sleep time with SpO_2_ < 90% (T90) and minimum SpO_2_ during sleep were derived from analysis of the entire diagnostic portion of the PSG, regardless of whether they were split-night or full-night studies.

Spindle density, power, frequency and %-fast in N2 sleep, and all qEEG metrics were averaged across the C3 and C4 electrodes. If data from one electrode was not available, values from the contralateral electrode were used.

### Statistical analyses

Primary analyses included all participants who had ≥60 minutes of total sleep time (TST) for digital analyses, and in whom age, sex, AHI, mean SpO_2_, T90, MoCA total score, and education status were available. Secondary analyses included only participants who underwent full-night PSG upon which digital analyses were performed on the entire record. Analyses were performed in R software (v4.2.2, The R Foundation for Statistical Computing, Vienna, Austria) with alpha set a priori at ≤0.05. All results reported are from the primary analyses, unless stated otherwise.

Differences in participant demographics, cognitive scores, conventional sleep metrics, spindle characteristics, ORP, and qEEG measures across OSA groups were determined using 1-way ANOVAs for normally distributed continuous variables (reported as mean ± SD) and Kruskall–Wallis tests for non-normally distributed continuous variables (reported as median [interquartile range]). Post hoc group comparisons incorporated either a Tukey or a Dwass, Steel, or Critchlow-Fligner correction, respectively. Fisher exact tests were used to compare categorical variables across groups incorporating a Bonferroni correction for post hoc comparisons.

For all subsequent analyses, any missing data were assumed to be missing at random and handled using multivariate imputation by chained equations based on the fully conditional specification as implemented in the *mice* package (v3.16.0) [54]. The only exceptions were missing RAVLT delayed recall and DSC z-scores, which were not imputed. Continuous and ordinal variables were imputed using propensity mean matching and the proportional odds model, respectively, while categorical variables were imputed using logistic regression. Auxiliary variables were included in the model to help reduce bias and improve the plausibility that data were missing at random [55]. A total of 50 imputed data sets were created on which analyses were performed independently, and the results were pooled.

Associations between spindle characteristics (density, power, frequency, and %-fast), ORP (ORP_TRT_, ORP_WAKE_, and ORP_NREM_), and qEEG measures (EEG_NP_ and D/A ratio) and cognitive scores were determined using multiple linear regression (MLR) adjusting for age, sex, education (except MoCA total score which includes an adjustment for low education; details in Supplementary Material), and TST. An age adjustment was included in MLR analyses of RAVLT delayed recall and DSC z-scores because the normative values employed to convert their raw scores to z-scores were for relatively large age bins (e.g. 18–49, 50–59, 60–69, etc.), which can result in potential residual confounding of age within regression models. To account for multiple hypothesis testing, a false discovery rate-adjusted *p*-value (*q*-value) of ≤ .05 was considered significant, with each group of associations (i.e. spindle characteristics, ORP, and qEEG measures) being treated independently. For all MLR analyses, normality of residuals, homoscedasticity, and multicollinearity assumptions were assessed by reviewing residual histograms (and residual-quantile plots), residual-predicted value plots, and the variance inflation factor, respectively.

Next, to identify potential mediators of the effect of moderate-to-severe OSA on cognitive dysfunction, parallel multiple mediation analyses were performed using the PROCESS macro (v4.3) [56]. Participants were first categorized into no/mild and moderate/severe OSA groups with the no/mild group acting as the reference. Next, propensity score matching was implemented with the *MatchIt* (v4.5.4) [57] and *MatchThem* (v1.1.0) [58] to match participants between the no/mild and moderate/severe OSA groups by age, sex, body mass index (BMI), education, ESS, PSQI, ISI, presence of RLS, smoking, comorbidities and medications across all imputed data sets. Adequate matching was defined as a standard mean difference for continuous variables and an absolute mean difference for categorical variables of ≤0.1. Potential mediators considered for inclusion in the final models were measures of hypoxemia (T90), sleep fragmentation (arousal-awakening index), sleep depth (ORP_TRT_, ORP_WAKE_, and ORP_NREM_), spindle activity (density, power, frequency, and %-fast), and overall EEG activity (EEG_NP_ and D/A ratio). Mediation analysis was performed for each cognitive outcome in independent models with potential mediators entered into the final models if they were associated with moderate/severe OSA and with cognitive scores controlling for only OSA group with a *p* ≤ .05 [59, 60]. Thus, the inclusion criteria for a variable to be considered a potential mediator was more liberal than the adjustments applied to the MLR analyses and determination of potential mediators to include in the final models was not based upon the MLR analyses. Mediation analyses were performed on each of the 50 imputed datasets independently using 10 000 bootstrap iterations per imputed data set with potential mediators entered as continuous variables with their mediation effects and 95% bias-corrected and accelerated confidence intervals estimated by pooling the results from all 500 000 bootstrap iterations. Collinearity between potential mediators was not assessed because in parallel multiple mediation analyses the impact of collinearity between mediators is minimized as no mediator is modeled as influencing any other in the model [56]. The AHI was not included as a potential mediator because it was used to categorize participants into OSA groups.

### Data availability

Anonymized data will be made available to qualified researchers on reasonable request to the corresponding author.

## Results

The CSCN cohort includes 2169 participants of which 1142 participants were included in our primary analyses (Table 1). Reasons for exclusion included not undergoing PSG (*n* = 521), PSGs not undergoing (*n* = 158) or failing (*n* = 28) digital analyses, TST ≤ 60 minutes (*n* = 113), and absence of a MoCA total score (*n* = 207). The entire cohort was predominantly male, middle-aged (range: 19–89 years), white individuals with moderate OSA and poor sleep quality. Hypertension, high cholesterol, and diabetes were the predominant comorbidities with statins and antidepressants the most used medications. Moderate and severe OSA groups included more males, who were older and had a higher BMI compared to the no/mild group (all comparisons, *p* ≤ .036). The severe OSA group had a higher ESS, greater proportion of participants with an ESS > 10, and a greater prevalence of subjective short sleep compared to both the no/mild and moderate groups (all comparisons, *p* ≤ .008). Finally, participants with moderate and severe OSA had a greater prevalence of past smokers, hypertension, high cholesterol, diabetes, coronary artery disease, and statin use (all post hoc comparisons, *p* ≤ .030 versus no/mild OSA group).

Cognitive scores are shown in Supplementary Table S1. The MoCA total score for the entire cohort was 25.6 ± 3.0 and decreased from 26 ± 2.9 to 25.1 ± 3.0 with increasing OSA severity; the severe OSA group had the lowest score and greatest prevalence of participants with scores < 26 (the validated score to indicate mild cognitive impairment [38]; all comparisons, *p* ≤ .019). The mean RAVLT delayed recall z-score was −0.3 ± 1.1 for the entire cohort, −0.2 ± 1.1 for participants with no/mild OSA, −0.3 ± 1.2 for moderate OSA, and −0.3 ± 1.1 for severe OSA; all z-scores were lower than zero (all comparisons, *p* < .001), indicating worse performance compared to age-matched normative values. However, scores were similar across OSA groups (*p* = .457). Similarly, mean DSC z-scores for the entire cohort (−0.6 ± 0.9), no/mild OSA (−0.5 ± 1.0), moderate OSA (−0.6 ± 1.0), and severe OSA (−0.7 ± 0.9) were lower than zero (all comparisons, *p* < .001) with the severe OSA group performing worse than the no/mild group (*p* = .016).

Table 2 shows conventional sleep metrics and ORP derived from the semi-automated MSS software. Compared to the no/mild OSA group, participants with moderate and severe OSA underwent a higher proportion of split-night PSGs, had a greater amount of NREM stage 1 (N1) and less NREM stage 3 (N3) sleep, and a higher arousal/awakening index (all post hoc comparisons, *p* ≤ .035); participants with severe OSA also had shorter total recording time (TRT) and TST, and lower sleep efficiency (all comparisons, *p* ≤ .012 vs. no/mild OSA). Compared to participants with moderate OSA, participants with severe OSA also had shorter TRT and TST, more N1 and less N2, N3, and REM sleep, and a higher arousal/awakening index (all comparisons, *p* ≤ .001). Finally, participants with severe OSA had a higher ORP_TRT_ and ORP_NREM_ compared to both the no/mild and moderate OSA groups (all comparisons, *p* ≤ .001).

**Table 2.** Conventional Sleep Metrics and the Odds Ratio Product (ORP) for the Entire Cohort and Categorized by OSA Severity^#^

	Entire cohort	No/Mild OSA	Moderate OSA	Severe OSA
*N*	1142	443	257	442
Split night PSG, *n* (%)	581 (50.9)	104 (23.5)	127 (49.4)^*^	350 (79.2)^*†^
Total recording time (min)	205.1 ± 59.9	219.4 ± 47.8	219.7 ± 59.3	182.3 ± 63.9^*†^
Total sleep time (TST; min)	146.0 ± 53.3	160.7 ± 47.9	157.2 ± 54.7	124.7 ± 50.8^*†^
Sleep efficiency (%)	74.7 (61.5–84.5)	76.1 (64.5–85.6)	73.9 (62.5–84.2)	73.7^*^ (57.2–83.4)
N1 (% TST)	16.8 (9.8–27.3)	11.0 (7.2–17.4)	16.2^*^ (10.7–23.3)	25.9^*†^ (16.4–41.9)
N2 (% TST)	60.2 (48.9–70.4)	62.4 (51.0–72.3)	61.6 (50.5–71.0)	56.8^*†^ (44.7–67.9)
N3 (% TST)	7.5 (0.0–22.9)	13.6 (2.2–30.6)	8.9^*^ (0.6–24.3)	1.5^*†^ (0.0–13.9)
REM (% TST)	0.0 (0.0–10.5)	3.0 (0.0–11.6)	2.0 (0.0–11.3)	0.0^*†^ (0.0–7.5)
Arousal/awakening index (/h)	32.5 (23.3–44.8)	25.4 (19.6–32.7)	31.1^*^ (23.6–39.6)	44.9^*†^ (33.8–63.9)
*Odds-ratio product*				
ORP_TRT_	1.28 (1.05–1.52)	1.18 (0.95–1.42)	1.20 (1.02–1.44)	1.41^*†^ (1.19–1.62)
ORP_WAKE_	2.17 (2.08–2.25)	2.18 (2.08–2.25)	2.17 (2.09–2.27)	2.17 (2.06–2.25)
ORP_NREM_	0.90 (0.71–1.12)	0.78 (0.64–0.98)	0.84 (0.69–1.00)	1.07^*†^ (0.86–1.27)

^#^values derived from analysis of the first half of all full-night PSGs and the diagnostic portion of all split-night PSGs.

Mean ± SD or median (interquartile range); ^*^p ≤ .05 versus No/Mild OSA; ^†^*p* ≤ .05 versus Moderate OSA.

*Abbreviations:* PSG, polysomnography; TST, total sleep time; N1, stage 1 non-rapid eye movement (NREM) sleep; N2, stage 2 NREM sleep; N3, stage 3 NREM sleep; ORP_TRT_, odds-ratio product for total recording time; ORP_WAKE_, odds-ratio product during wakefulness; and ORP_NREM_, odds ratio product during NREM sleep.

Compared to participants with no/mild OSA, spindle density, power, frequency, and %-fast in N2 sleep were lower in participants with moderate and severe OSA (all comparisons, *p* ≤ .001; Table 3). Spindle density was also lower in participants with severe OSA compared to those with moderate OSA (*p* = .003). The moderate and severe OSA groups had lower absolute EEG power in the fast delta + theta (2.67–6.33 Hz) and sigma (12.33–14.0 Hz) frequency bands compared to the no/mild OSA group (all post hoc comparisons, *p* ≤ .037) while the severe OSA group also had lower absolute alpha (7.33–12.0 Hz) power (*p* = .036, vs. no/mild OSA). In addition, participants with severe OSA had lower relative power in the fast delta + theta band and higher absolute powers in the beta-1 (14.33–20.0 Hz), beta-2 (20.33–35.0 Hz), and gamma (35.3–60.0 Hz) bands compared to the no/mild and moderate OSA groups, and higher relative power in the beta-1 and beta-2 frequency bands compared to no/mild group (all post hoc comparisons, *p* ≤ .051). The D/A ratio was not different between groups (*p* = .191).

**Table 3.** Spindle Characteristics, Power Spectrum and Quantitative EEG (qEEG) Metrics for the Entire Cohort and Categorized by OSA Severity

	Entire cohort	No/Mild OSA	Moderate OSA	Severe OSA
*Spindles*				
*N2 sleep*				
*N*	1,142	443	257	442
*Density (/min)*	2.5 ± 1.5	2.9 ± 1.7	2.5 ± 1.4^*^	2.1 ± 1.3^*†^
*Power (uV²)*	29.5 ± 10.8	31.9 ± 11.9	28.9 ± 9.3^*^	27.4 ± 10.0^*^
*Frequency (Hz)*	12.0 ± 0.6	12.1 ± 0.5	11.9 ± 0.6^*^	11.9 ± 0.6^*^
*% Fast*	47.6 ± 21.0	52.3 ± 20.0	44.0 ± 21.3^*^	45.0 ± 21.0^*^
*EEG power spectrum*				
*Total power*				
*uV* ^*2*^	91.45 (64.91–138.31)	94.35 (66.40–140.53)	90.90 (64.35–130.84)	90.65 (62.22–146.07)
*EEG*_*NP*_	0.87 (0.67–1.19)	0.89 (0.67–1.23)	0.83 (0.65–1.13)	0.87 (0.68–1.16)
*Slow delta (0.33–2.33Hz)*				
*uV* ^*2*^	65.00 (42.00–104.91)	66.00 (43.00–104.07)	62.25 (40.95–99.00)	65.00 (41.05–109.50)
*Relative (%)*	70.5 (63.7–77.0)	70.2 (64.2–75.9)	70.0 (64.3–76.1)	71.6 (63.0–78.8)
*Fast delta±theta (2.67–6.33Hz)*				
*uV* ^*2*^	12.35 (9.20–16.65)	13.55 (10.07–18.83)	12.53^*^ (8.95–16.55)	11.40^*†^ (8.35–15.30)
*Relative (%)*	13.7 (11.3–16.0)	14.3 (12.1–16.6)	14.0 (11.7–16.0)	12.7^*†^ (10.4–14.9)
*Alpha (7.33–12.0Hz)*				
*uV* ^*2*^	7.20 (5.00–11.05)	7.50 (5.20–11.45)	7.50 (4.95–11.10)	6.82^*^ (4.81–10.07)
*Relative (%)*	8.0 (5.8–11.3)	8.1 (6.0–11.1)	8.4 (6.1–11.2)	7.8 (5.1–11.7)
*Sigma (12.33–14.0Hz)*				
*uV* ^*2*^	1.30 (0.95–1.80)	1.35 (1.00–2.00)	1.25^*^ (0.95–1.70)	1.20^*^ (0.90–1.70)
*Relative (%)*	1.4 (1.0–2.0)	1.5 (1.1–2.0)	1.4 (1.0–1.9)	1.4 (1.0–1.9)
*Beta-1 (14.33–20.0Hz)*				
*uV* ^*2*^	1.98 (1.55–2.70)	1.90 (1.45–2.70)	1.85 (1.50–2.60)	2.05^*†^ (1.65–2.75)
*Relative (%)*	2.2 (1.5–3.2)	2.1 (1.4–2.9)	2.2 (1.5–3.1)	2.4^*^ (1.6–3.4)
*Beta-2 (20.33–35.0Hz)*				
*uV* ^*2*^	1.70 (1.20–2.55)	1.60 (1.15–2.38)	1.65 (1.15–2.35)	1.95^*†^ (1.30–2.70)
*Relative (%)*	1.9 (1.2–3.0)	1.7 (1.1–2.6)	1.9 (1.2–3.0)	2.0^*^ (1.3–3.2)
*Gamma (35.3–60.0Hz)*				
*uV* ^*2*^	0.45 (0.30–0.75)	0.42 (0.30–0.74)	0.45 (0.30–0.70)	0.50^*†^ (0.32–0.80)
*Relative (%)*	0.5 (0.3–0.9)	0.4 (0.3–0.8)	0.5 (0.3–0.9)	0.5 (0.3–0.9)
*qEEG measures*				
D/A ratio	8.7 (5.6–13.4)	8.6 (5.8–12.6)	8.3 (5.7–12.2)	9.1 (5.3–15.6)

Mean ± SD or median (interquartile range); ^*^p ≤ .05 versus No/Mild OSA; ^†^*p* ≤ .05 versus Moderate OSA.

*Abbreviations:* N2, stage 2 non-rapid eye movement (NREM) sleep; D/A ratio, ratio between the delta and alpha power spectrum bands.

**Figure 1.**
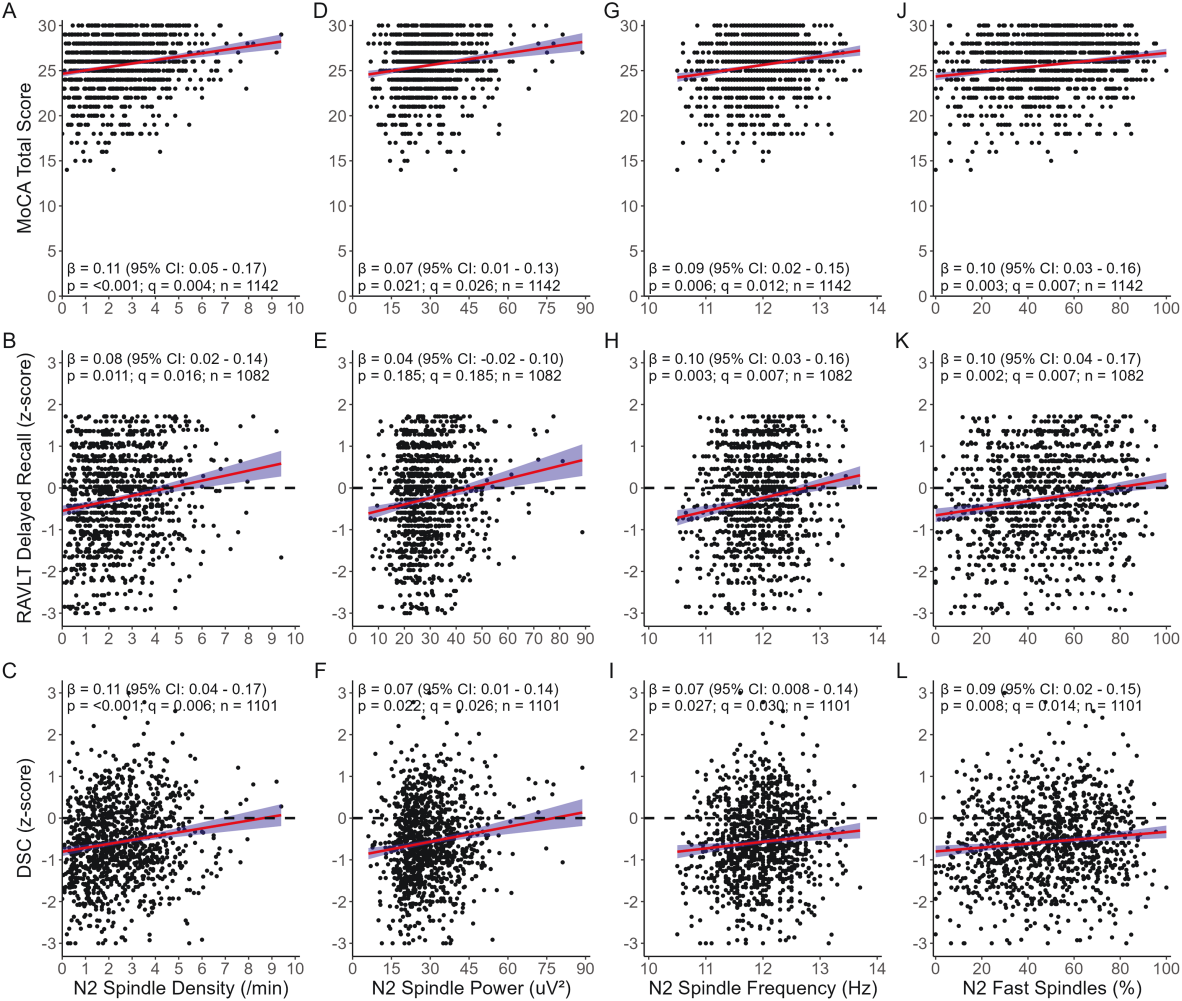
Linear associations between cognitive scores and N2 spindle density (A-C), power (D-F), frequency (G-I), and percentage of fast spindles (J-L) in our *primary analysis cohort* of participants with ≥60 minutes of total sleep time. Standardized parameter estimates (β) and 95% CI provided in plots reflect the relationships between cognitive scores and spindle characteristics adjusting for age, sex, education (except MoCA), total sleep time, and OSA severity group. *Abbreviations:* DSC, Weschler Adult Intelligence Scale 4th Edition WAIS-IV digit symbol coding; MoCA, Montreal cognitive assessment; RAVLT, Rey auditory verbal learning test.

**Figure 2.**
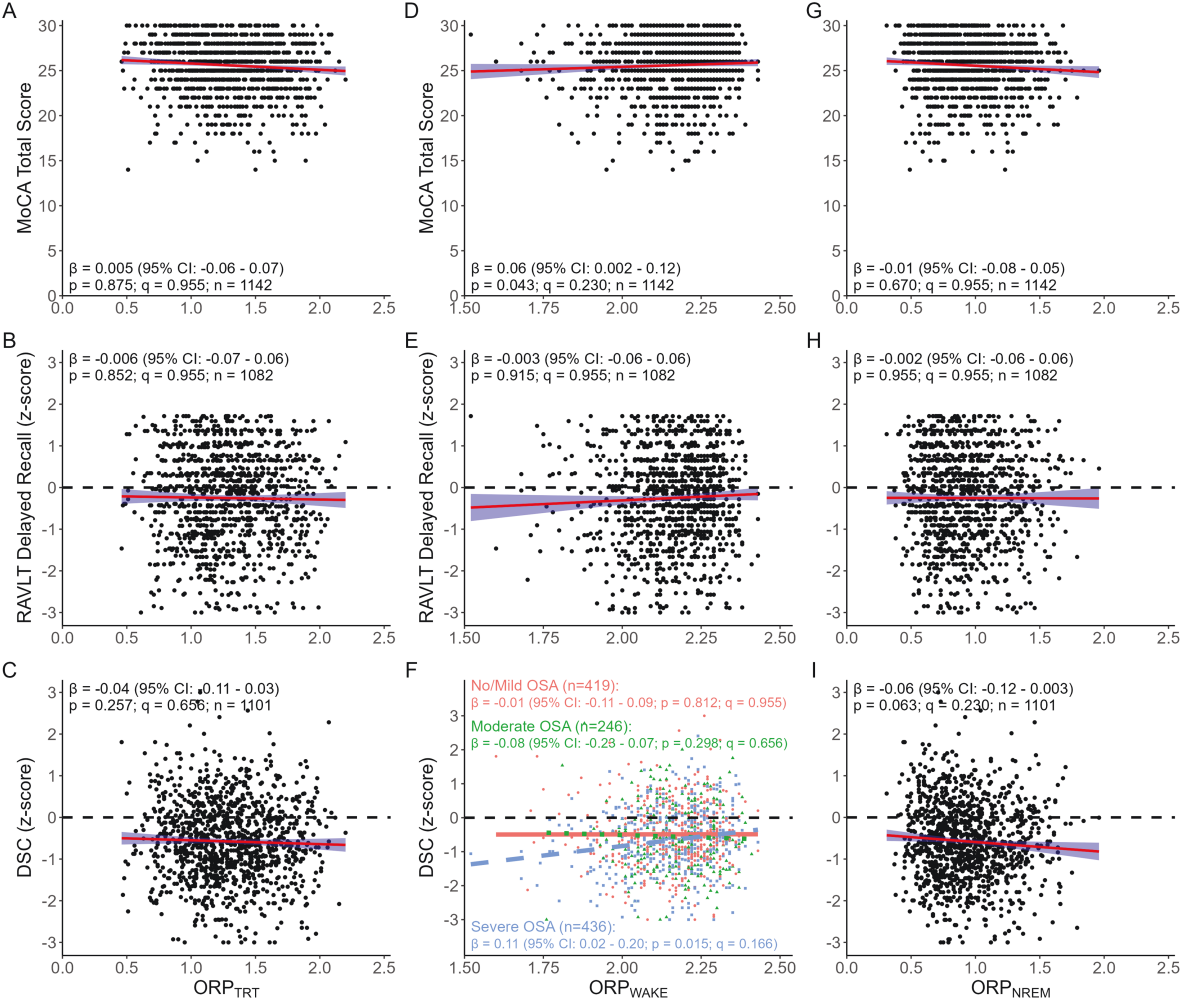
Linear associations between cognitive scores and the odds-ratio product for total recording time (ORP_TRT_; A-C), during wakefulness (ORP_WAKE_; D-F), and during non-rapid eye movement sleep (ORP_NREM_; G-I) in our *primary analysis cohort* of participants with ≥60 minutes of total sleep time. Standardized parameter estimates (β) and 95% CI provided in plots reflect the relationships between cognitive scores and ORP adjusting for age, sex, education (except MoCA), total sleep time, and OSA severity group. Associations between DSC and ORP_WAKE_ are reported for each group (F) because of a significant group-by-ORP_WAKE_ interaction (No/Mild OSA = solid line; Moderate OSA = dotted line; and Severe OSA = dashed line). *Abbreviations:* DSC, Weschler Adult Intelligence Scale 4th Edition WAIS-IV digit symbol coding; MoCA, Montreal cognitive assessment; RAVLT, Rey auditory verbal learning test.

For MLR analyses, the interaction between all spindle characteristics, ORP_TRT_, ORP_NREM_, EEG_NP_, and the D/A ratio index and OSA group was not significant for any cognitive score (all interactions, *p* ≥ .054). Therefore, associations with cognitive scores were quantiﬁed using all participants but controlling for age, sex, education (except MoCA), TST, and OSA group. For ORP_WAKE_ the interaction with the OSA group was significant for DSC (*p* = .021); therefore, associations were reported for each group. Spindle density, frequency, and %-fast were positively associated with all cognitive test scores (all associations, *q* ≤ 0.03) while spindle power was positively associated with the MoCA total and DSC scores (both associations, *q* = 0.026), but not RAVLT delayed recall (*q* = 0.185; Figure 1). However, in our secondary analyses of data from only participants who underwent full night PSG, associations between all cognitive scores and spindle density and power, and between DSC and spindle frequency and %-fast were no longer significant (*q* ≥ 0.077; Supplementary Figure S1). ORP measures were not associated with cognitive performance within our primary analyses (all associations, primary: *q* ≥ 0.166, Figure 2), but ORP_WAKE_ was positively associated with MoCA total score in our secondary analyses (*q* = 0.016, Supplementary Figure S3) analyses. Similarly, EEG_NP_ and the D/A ratio were not associated with cognitive scores in either analysis (all associations, *q* ≥ 0.088; Supplementary Table S1).

**Figure 3.**
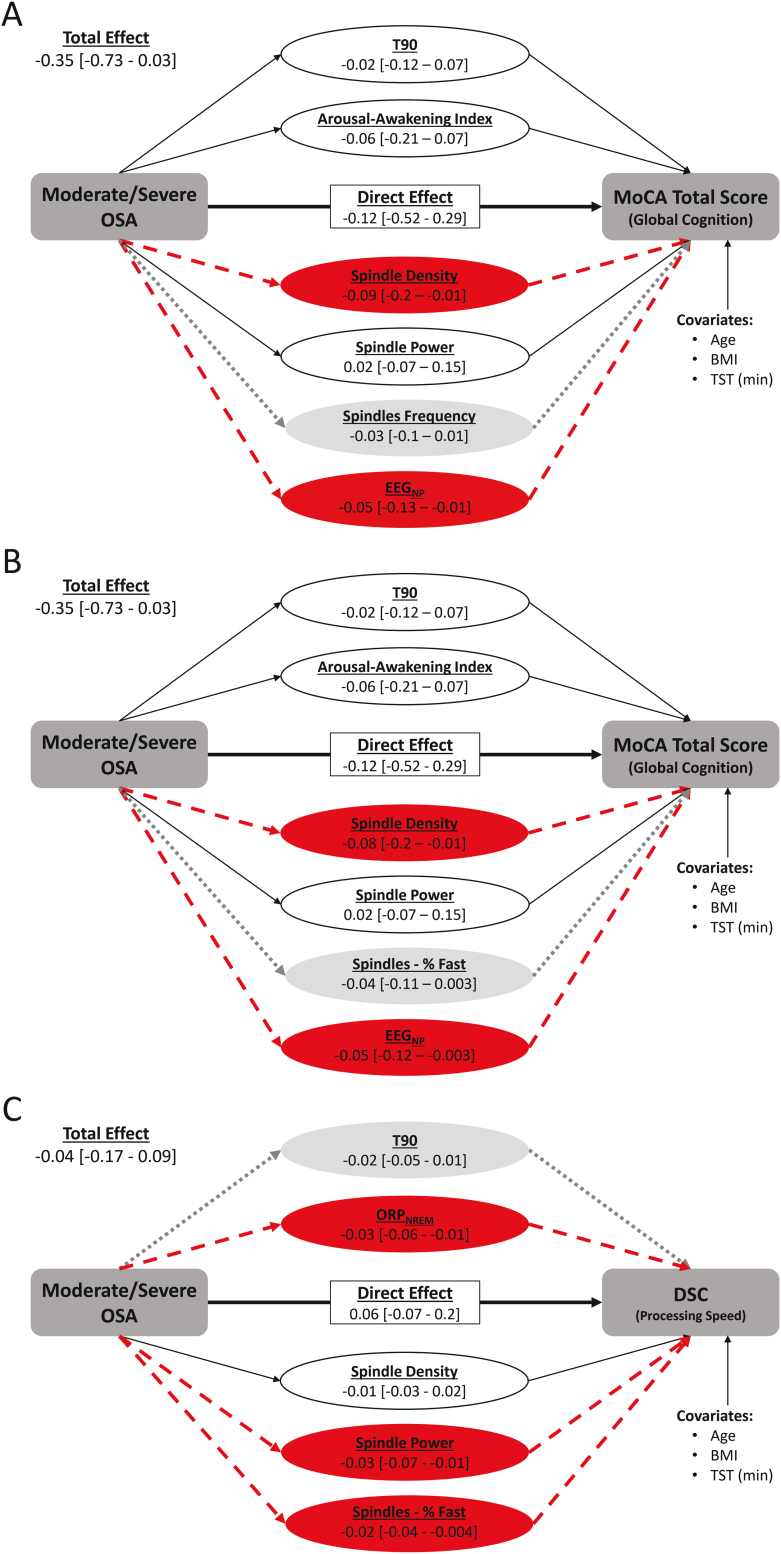
Mediation analyses assessing the effect of moderate/severe OSA on the Montreal cognitive assessment (MoCA) total score (A, spindle frequency included in the mediation model; B, spindles %-fast included in the mediation model) and the Weschler Adult Intelligence Scale 4th Edition (WAIS-IV) digit symbol coding (DSC) score (C) in our *primary analysis cohort* of participants with ≥60 minutes of total sleep time. Values are the mean effects and 95% bias-corrected and accelerated confidence intervals pooled across all 50 imputed data sets (10 000 bootstrap iterations per data set). Significant mediators are shown with dashed arrows while potential mediators whose 95% confidence interval just failed to exclude zero are shown with dotted arrows; nonsignificant mediators are shown with solid black arrows. *Abbreviations:* % Fast, percentage of spindles with a frequency of 12–16 Hz; BMI, body mass index; EEG_NP_, overall normalized EEG power; ORP_NREM_, odds-ratio product during non-rapid eye movement sleep; T90, percentage of total sleep time with oxyhemoglobin saturation <90%; and TST, total sleep time.

Since RAVLT delayed recall scores were similar across OSA groups in our primary analyses, mediation analysis was performed for only MoCA total and DSC scores. Following propensity score matching, 1107 participants (moderate/severe OSA, *n* = 664) were included in our primary mediation analyses for the MoCA and 1047 (moderate/severe OSA, *n* = 628) were included in our primary mediation analyses for the DSC. Secondary analyses included 607 (moderate/severe OSA, *n* = 204) and 490 (moderate/severe OSA, *n* = 204) participants for the MoCA and DSC, respectively. For all mediation models, in addition to TST, age and BMI were included as covariates because they could not be adequately matched between OSA groups (Supplementary Figure S3). For the MoCA total score, potential mediators entered in the final model were T90, the arousal-awakening index, all spindle characteristics (density, power, frequency, and %-fast), and EEG_NP_. Due to the high correlation between spindle frequency and %-fast (*r* = 0.97), separate models were run for each (Figure 3, A and B). In both models, spindle density and EEG_NP_ were the only mediators of the negative effect of moderate/severe OSA on the MoCA total score, although the 95% confidence intervals for spindle frequency and %-fast just failed to exclude zero. In our secondary analyses the arousal-awakening index along with spindle frequency and %-fast spindles were mediators of the negative effect of moderate/severe OSA on the MoCA total score (Supplementary Figure S4A and B); however, the 95% confidence interval for T90 just failed to exclude zero. For the DSC, potential mediators included in the final model were T90, ORP_NREM_, and spindle density, power, and %-fast. Of these, ORP_NREM_, spindle power, and the %-fast spindles were mediators of the negative effects of moderate/severe OSA on DSC performance within our primary analyses while the 95% confidence interval for T90 just failed to exclude zero (primary, Figure 3C). In our secondary analyses, results were comparable with %-fast spindles being a mediator of the negative effect of moderate/severe OSA on DSC performance with the 95% confidence interval for T90, ORP_NREM_ and spindle power just failing to exclude zero (Supplementary Figures S4C).

## Discussion

Using PSG data from a large cohort of sleep clinic patients with OSA and a high prevalence of cognitive impairment [10], this study investigated the associations between cognitive function and measures of sleep microarchitecture, and determined if measures of sleep microarchitecture mediate the relationship between moderate-to-severe OSA and lower cognitive performance. The main findings were that (1) compared to participants with no/mild OSA, patients with moderate and severe OSA had reduced spindle density, power, frequency, and percentage of fast spindles; (2) lower measures of global cognitive function (MoCA) and information processing speed (DSC) were associated with lower spindle density, power, frequency and percentage of fast spindles independent of age, sex, education, and TST while worse memory (RAVLT delayed recall) was associated with lower spindle density, frequency and percentage of fast spindles; and (3) in mediation models that included measures of nocturnal hypoxia (T90) and/or sleep fragmentation (arousal-awakening index), spindle density and EEG_NP_ were mediators of the negative impact of moderate-to-severe OSA on the MoCA total score, while ORP_NREM_, spindle power and the percentage of fast spindles mediated the negative impact of moderate-to-severe OSA on information processing speed. These results indicate that OSA-related changes in sleep microarchitecture, particularly reduced spindle activity, may be important contributors to cognitive deficits in patients with OSA.

Sleep spindles are short, oscillatory bursts of EEG activity of 10–16 Hz that last between 0.5 and 3 seconds and are a defining characteristic of N2 sleep. Although results are discordant between studies investigating the impact of OSA on spindle activity, the current study’s findings of lower spindle activity with increasing OSA severity align with several prior small studies (*n* ≤ 36) that report patients with OSA have lower N2 spindle density, power, frequency, and percentage of fast spindles [25, 61–63]. Moreover, a large population-based multicohort study (*n* = 9883) [64] and a population-based male cohort study (*n* = 675) [65] both found participants with severe OSA (AHI ≥ 30) had lower N2 spindle density compared to individuals with no/mild OSA. Although, in contrast to the current study where moderate and severe OSA was associated with both lower N2 spindle density and frequency compared to participants with no/mild OSA, the male cohort study found participants with severe OSA had higher spindle frequency compared to the no/mild OSA group (AHI < 10) [65]. On the other hand, several small studies (*n* ≤ 54) report no difference in N2 spindle density [66, 67] or spindle power [68, 69] between patients with, and without, OSA; one study also found higher spindle power in participants with OSA [70]. Reasons for the conflicting findings across studies are unclear. Methodological differences in spindle detection and spindle bandwidths employed between studies may have contributed [24], but differences in study cohorts (population vs. sleep clinic-based) are likely a greater contributor, particularly given the high prevalence of poor sleep quality and cognitive impairment within the sleep clinic cohort employed in the current study (Tables 1 and Supplementary Table S1); traits that are associated with reduced spindle activity [71, 72].

Although the function of sleep spindles is incompletely understood, they are thought to reflect the strength of thalamocortical circuits essential to cognition with increased spindle activity being important for memory consolidation, learning, cognitive abilities underlying executive functions, and overall intelligence [18, 64, 71, 73, 74]. Nonetheless, no studies have investigated the potential role of reduced spindle activity on measures of global cognition, memory, and information processing speed in adult sleep clinic patients with OSA. Instead, the small studies (*n* = 8–24) that have investigated the relationship between spindle activity and cognition in OSA have focused on overnight learning of motor skills [75], statistical (implicit) learning [76], memory consolidation [77], and vigilance [25]. In general, these studies found greater spindle activity was associated with better cognitive performance. Specifically, positive correlations were observed between spindle density and overnight improvement of a single finger tapping task [75], spindle frequency and statistical learning [76], and fast (12–15Hz) spindle density and memory retention [77]. Additionally, negative correlations were observed between spindle density and reaction time [25]. As such, the positive associations between spindle characteristics and cognitive scores found in the current study (Figure 1) align with these earlier studies, thus reflecting the importance of spindle activity on cognitive function in patients with OSA.

The predominant features of OSA thought to contribute to cognitive impairment are nocturnal hypoxemia and sleep fragmentation [12, 13] even though the strength of the relationships between these conventional indices of OSA severity and cognitive function using linear regression are modest [10, 13–16]. In addition to assessing associations between cognitive function and measures of sleep microarchitecture using multiple linear regression the current study also performed parallel mediation analyses to investigate whether moderate-to-severe OSA exerted its detrimental effect on cognitive function through nocturnal hypoxemia, sleep fragmentation, and changes in sleep microarchitecture. For these analyses, variables considered as potential mediators included T90 (conventional measure of nocturnal hypoxemia), the arousal-awakening index (conventional measure of sleep fragmentation), ORP (averaged across TRT, wakefulness and NREM sleep; novel measure of sleep depth), and spindle activity (density, power, frequency, and %-fast) along with EEG_NP_ and the D/A ratio. To be included in the final mediation models, variables needed to be both correlated with moderate/severe OSA and associated with cognitive scores independent of OSA group with a *p* ≤ .05 [59,60]. These criteria were independent of MLR results because, in contrast to MLR analyses which were used to investigate associations between cognitive scores and EEG measures of sleep microarchitecture adjusting for the differences in mean values of cognitive scores and EEG measures between OSA severity groups, mediation analyses was used to investigate which potential pathways may contribute to the lower cognitive scores observed between participants with moderate-severe OSA and no/mild OSA. Similar to earlier findings of associations between cognitive function and various measures of nocturnal hypoxemia, but not sleep fragmentation [78, 79], in the current study T90 met these inclusion criteria for both the MoCA and DSC while the arousal-awakening index only met these criteria for the MoCA, results suggesting nocturnal hypoxemia may be a more important contributor to cognitive impairment in OSA than sleep fragmentation. In contrast to T90 and the arousal-awakening index, all spindle characteristics met the required criteria to be included in the final mediation models examining how moderate-to-severe OSA negatively impacts MoCA and DSC scores. Furthermore, neither T90 nor the arousal-awakening index were significant mediators of moderate-to-severe OSA’s detrimental effect on either cognitive measure. Rather, spindle density and EEG_NP_ emerged as mediators of the negative effect of moderate-to-severe OSA on global cognitive function (Figure 3, A and B) while spindle power and ORP_NREM_ mediated the negative impact of moderate-to-severe OSA on information processing speed (Figure 3C). These results suggest that OSA-related disruption of sleep microarchitecture, particularly spindle activity, may be a principal pathway through which moderate-to-severe OSA exerts its harmful effects on cognitive function.

This study has several strengths. First, it was performed using data from a large, multi-center cohort of sleep clinic patients with a high prevalence of considerable OSA and cognitive impairment, and the changes in sleep microarchitecture in patients with moderate and severe were compared to a clinically relevant control group of sleep clinic patients with no or mild OSA. Both features improve the generalizability of our findings to patients with moderate and severe OSA. Second, detailed clinical phenotyping enabled us to control for confounding variables in our assessment of relationships between cognitive function and measures of sleep microarchitecture and to propensity score match participants prior to mediation analyses. Third, the measures of sleep macro- and microstructure were comprehensive and derived using validated analyses [18, 41–43] performed by an investigator blinded to the cognitive results.

This study also has limitations. First, in our primary analysis cohort, 50% of participants underwent split-night PSG. To maximize the sample size, digital analyses of full-night PSGs were limited to the first half of the PSG study to align temporally with the diagnostic portion of the split-night PSGs and participants were included if they had ≥60 minutes of TST. While ≥60 minutes of TST is relatively short for the diagnosis of OSA, it enabled us to include participants with a broad range of OSA severity; in contrast to the 619 participants in our secondary analyses, of whom 64% had no/mild OSA. Furthermore, among the 561 participants in our primary analysis cohort who underwent full-night PSG, a comparison of the AHI derived from the 1^st^ half of the night to the AHI derived from the entire PSG resulted in 118 participants changing OSA severity group, of whom 83 moved to a lower OSA severity. Furthermore, of these 118 participants, 90 had a TST ≥120 minutes during the first half of the night, a sleep duration previously reported to have a 97% sensitivity and 82.8% specificity to detect an AHI of 25 [80]. Although the same comparison cannot be made for the 581 participants who underwent split night PSGs in our primary cohort, a longer TST is unlikely to alter the OSA severity group to which they were assigned, since most of them had moderate and severe OSA. Consequently, we believe that the categorization of participants by OSA severity (i.e. no/mild, moderate, and severe) was accurate in our primary analyses’ cohort, which is the variable used in the multiple linear regression and mediation analyses. One consequence of using split-night PSGs was that the duration of REM sleep was too short for analyses of sleep microarchitecture and qEEG measures isolated to this sleep stage (Table 2). Similarly, analyses of REM sleep were not performed in our secondary sensitivity analyses of participants who underwent full-night PSG because only 70% of participants (433 out of 619) had a sufficient duration of REM sleep for robust analyses (defined as ≥30 minutes [81]), of which 69% (298 out of 433) had no/mild OSA who are not at increased risk of cognitive impairment, and only 35 participants had severe OSA who are at greatest risk of cognitive impairment [10]. Importantly, results were similar between our primary and secondary analyses for associations between MoCA total and RAVLT delayed recall scores and spindle frequency and %-fast; and although the associations between cognitive scores and spindle density and power were no longer significant, similar positive relationships were observed between spindle density and the MoCA total and DSC scores which just failed to reach statistical significance after correcting for the false discovery rate (*q* = 0.079 and 0.076, respectively). In mediation analyses, our secondary analyses found comparable results to our primary analyses for how moderate-to-severe OSA exerts its negative effects on DSC scores although the 95% confidence intervals for ORP_NREM_ and spindle power just failed to exclude zero, while the arousal-awakening index and spindle frequency were significant mediators of the negative effect of moderate-to-severe OSA on the MoCA total score. These differences likely resulted from the removal of 49% of participants with moderate OSA and 79% of participants with severe OSA who underwent split-night PSG (Table 2), thereby implying sleep fragmentation may be a more important mechanism through which milder OSA negatively impacts global cognitive function. Second, the parallel mediation analyses employed may not capture the complexity of the causal pathway through which OSA exerts its negative effects on cognitive function whereby mediators may act in serial and/or interactive ways. In addition, our mediation model is not exhaustive and other measures of sleep microarchitecture that can be altered by OSA that were not included in our analyses such as during REM sleep (e.g., EEG slowing index [82]), the wake intrusion index [83], the ORP-9 [84] and ORP architecture [85] may also mediate some of the negative effects of moderate/severe on cognitive function. Third, the 95% confidence intervals for the total effect of some mediation models included zero (i.e. were not statistically significant). However, a significant total effect was not considered a prerequisite for reporting the mediation results because a) even with a nonsignificant total effect it is still possible for a portion of a predictor’s effect (i.e. moderate/severe OSA) on the outcome variable (i.e. MoCA total and DSC scores) to be partly conveyed through a mediator pathway [56, 86, 87]; and b) the scenario of a nonsignificant total effect, but significant mediation effects is more common in multiple mediation analyses such as performed in the current study due to inconsistent/suppression mediation models occurring more frequently [88]. Fourth, spindle characteristics were derived from central EEG electrodes and averaged across the C3 and C4 electrode sites. As such, topographical differences in spindle characteristics between OSA severity groups and their potential associations with cognitive function, could not be investigated. Fifth, sleep duration was self-reported which is subjective and open to error [89]. An objective assessment of sleep duration with a methodology such as actigraphy would have been preferable for more optimal matching within the mediation analyses. Finally, the cross-sectional study design does not allow for causal inferences to be made between cognitive function and the observed changes in sleep microarchitecture.

### Clinical implications

The yearly cost of treating and caring for patients with dementia is ~$10 billion in Canada and ~$321 billion in the United States, making it not only a public health emergency, but an economic priority [90, 91]. Given OSA is an accepted risk factor for mild cognitive impairment (a symptomatic pre-dementia cognitive state) and dementia [6], it represents a modifiable risk factor for the prevention of these long-term health consequences and reduction of the personal and financial costs of these diseases upon patients, society, and health care systems. However, conventional measures of OSA severity and symptom subtypes do not adequately identify OSA patients with, and without, cognitive deficits [17]. The results of the current study suggest that OSA-related disruption of sleep microarchitecture, particularly spindle activity, are potential additional biomarkers to identify patients at the greatest risk of cognitive impairment. Future longitudinal studies are needed to assess the impact of OSA treatment on sleep microarchitecture and cognitive function to determine if treatment of OSA improves sleep microarchitecture concurrent with recovery of cognitive function or mitigation of further decline. As such, changes in sleep microarchitecture could be important inclusion criteria for the recruitment of OSA patients at risk of cognitive decline who may benefit the most from OSA treatment.

## Supplementary Material

Supplementary material is available at *SLEEP* online.

zsae141_suppl_Supplementary_Tables_S1-S2_Figures_S1-S4

## References

[1] Benjafield AV , Ayas NT, Eastwood PR, et al Estimation of the global prevalence and burden of obstructive sleep apnoea: a literature-based analysis. Lancet Respir Med. 2019;7(8):687–698. doi: https://doi.org/10.1016/S2213-2600(19)30198-5 31300334 PMC7007763

[2] Beaudin AE , Raneri JK, Ahmed SB, et al Risk of chronic kidney disease in patients with obstructive sleep apnea. Sleep. 2022;45(2). doi: https://doi.org/10.1093/sleep/zsab267 PMC8842337 34757390

[3] Beaudin AE , Waltz X, Hanly PJ, Poulin MJ. Impact of obstructive sleep apnoea and intermittent hypoxia on cardiovascular and cerebrovascular regulation. Exp Physiol. 2017;102(7):743–763. doi: https://doi.org/10.1113/EP086051 28439921

[4] Tasali E , Ip MSM. Obstructive sleep apnea and metabolic syndrome: alterations in glucose metabolism and inflammation. Proc Am Thorac Soc. 2008;5(2):207–217. doi: https://doi.org/10.1513/pats.200708-139MG 18250214

[5] Dempsey JA , Veasey SC, Morgan BJ, O'Donnell CP. Pathophysiology of sleep apnea. Physiol Rev. 2010;90(1):47–112. doi: https://doi.org/10.1152/physrev.00043.2008 20086074 PMC3970937

[6] Lal C , Ayappa I, Ayas N, et al The link between obstructive sleep apnea and neurocognitive impairment: An Official American Thoracic Society Workshop Report. Ann Am Thorac Soc. 2022;19(8):1245–1256. doi: https://doi.org/10.1513/AnnalsATS.202205-380ST 35913462 PMC9353960

[7] Angelelli P , Macchitella L, Toraldo DM, et al The neuropsychological profile of attention deficits of patients with obstructive sleep apnea: an update on the daytime attentional impairment. Brain Sci. 2020;10(6):325. doi: https://doi.org/10.3390/brainsci10060325 32471112 PMC7349097

[8] Bucks RS , Olaithe M, Eastwood P. Neurocognitive function in obstructive sleep apnoea: a meta-review. Respirology. 2013;18(1):61–70. doi: https://doi.org/10.1111/j.1440-1843.2012.02255.x 22913604

[9] Gagnon K , Baril A-A, Gagnon J-F, et al Cognitive impairment in obstructive sleep apnea. Pathol Biol (Paris). 2014;62(5):233–240. doi: https://doi.org/10.1016/j.patbio.2014.05.015 25070768

[10] Beaudin AE , Raneri JK, Ayas NT, et al Cognitive function in a sleep clinic cohort of patients with obstructive sleep apnea. Ann Am Thorac Soc. 2021;18(5):865–875. doi: https://doi.org/10.1513/AnnalsATS.202004-313OC 33147067

[11] Otero L , et al Cognitive impairment and obstructive sleep apnea. In: Rossi FH, et al, eds. Updates in Sleep Neurology and Obstructive Sleep Apnea. Rijeka, Croatia: IntechOpen; 2019:chap 9.

[12] Sforza E , Roche F. Sleep apnea syndrome and cognition. Front Neurol. 2012;3:87. doi: https://doi.org/10.3389/fneur.2012.00087 22661967 PMC3361858

[13] Quan SF , Chan CS, Dement WC, et al The association between obstructive sleep apnea and neurocognitive performance--the Apnea Positive Pressure Long-term Efficacy Study (APPLES). Sleep. 2011;34(3):303–314B. doi: https://doi.org/10.1093/sleep/34.3.303 21358847 PMC3041706

[14] Beebe DW. Neurobehavioral effects of obstructive sleep apnea: an overview and heuristic model. Curr Opin Pulm Med. 2005;11(6):494–500. doi: https://doi.org/10.1097/01.mcp.0000183059.52924.39 16217174

[15] Boland LL , Shahar E, Iber C, Knopman DS, Kuo TF, Nieto FJ; Sleep Heart Health Study (SHHS) Investigators. Measures of cognitive function in persons with varying degrees of sleep-disordered breathing: the Sleep Heart Health Study. J Sleep Res. 2002;11(3):265–272. doi: https://doi.org/10.1046/j.1365-2869.2002.00308.x 12220323

[16] Sanchez AI , Martínez P, Miró E, Bardwell WA, Buela-Casal G. CPAP and behavioral therapies in patients with obstructive sleep apnea: effects on daytime sleepiness, mood, and cognitive function. Sleep Med Rev. 2009;13(3):223–233. doi: https://doi.org/10.1016/j.smrv.2008.07.002 19201228

[17] Allen AJH , et al Symptom subtypes and cognitive function in a clinic-based OSA Cohort: A Multi-Centre Canadian Study. Sleep Med. 2020;74:92–98. doi: https://doi.org/10.1016/j.sleep.2020.05.001 32841852 PMC9680684

[18] Guadagni V , Byles H, Tyndall AV, et al Association of sleep spindle characteristics with executive functioning in healthy sedentary middle-aged and older adults. J Sleep Res. 2021;30(2):e13037. doi: https://doi.org/10.1111/jsr.13037 32281182

[19] Djonlagic I , Mariani S, Fitzpatrick AL, et al Macro and micro sleep architecture and cognitive performance in older adults. Nat Hum Behav. 2021;5(1):123–145. doi: https://doi.org/10.1038/s41562-020-00964-y 33199858 PMC9881675

[20] Mander BA , Rao V, Lu B, et al Impaired prefrontal sleep spindle regulation of hippocampal-dependent learning in older adults. Cereb Cortex. 2014;24(12):3301–3309. doi: https://doi.org/10.1093/cercor/bht188 23901074 PMC4224242

[21] Mander BA , Dave A, Lui KK, et al Inflammation, tau pathology, and synaptic integrity associated with sleep spindles and memory prior to beta-amyloid positivity. Sleep. 2022;45(9). doi: https://doi.org/10.1093/sleep/zsac135 PMC9758508 35670275

[22] Younes M , Redline S, Peters K, et al Normalized EEG power: a trait with increased risk of dementia. Sleep. 2023;46(12). doi: https://doi.org/10.1093/sleep/zsad195 PMC10710983 37471250

[23] D’Rozario AL , Cross NE, Vakulin A, et al Quantitative electroencephalogram measures in adult obstructive sleep apnea – Potential biomarkers of neurobehavioural functioning. Sleep Med Rev. 2017;36:29–42. doi: https://doi.org/10.1016/j.smrv.2016.10.003 28385478

[24] Weiner OM , Dang-Vu TT. Spindle oscillations in sleep disorders: a systematic review. Neural Plast. 2016;2016:7328725. doi: https://doi.org/10.1155/2016/7328725 27034850 PMC4806273

[25] Mullins AE , Kim JW, Wong KKH, et al Sleep EEG microstructure is associated with neurobehavioural impairment after extended wakefulness in obstructive sleep apnea. Sleep Breath. 2021;25(1):347–354. doi: https://doi.org/10.1007/s11325-020-02066-5 32772308

[26] Younes M , Azarbarzin A, Reid M, Mazzotti DR, Redline S. Characteristics and reproducibility of novel sleep EEG biomarkers and their variation with sleep apnea and insomnia in a large community-based cohort. Sleep. 2021;44(10). doi: https://doi.org/10.1093/sleep/zsab145 PMC8503837 34156473

[27] Wang D , Piper AJ, Yee BJ, et al Hypercapnia is a key correlate of EEG activation and daytime sleepiness in hypercapnic sleep disordered breathing patients. J Clin Sleep Med. 2014;10(5):517–522. doi: https://doi.org/10.5664/jcsm.3700 24910553 PMC4046358

[28] Wang J , Xu J, Liu S, et al Electroencephalographic activity and cognitive function in middle-aged patients with obstructive sleep apnea before and after continuous positive airway pressure treatment. Nat Sci Sleep. 2021;13:1495–1506. doi: https://doi.org/10.2147/NSS.S322426 34475793 PMC8407675

[29] Sivam S , Poon J, Wong KKH, et al Slow-frequency electroencephalography activity during wake and sleep in obesity hypoventilation syndrome. Sleep. 2020;43(2). doi: https://doi.org/10.1093/sleep/zsz214 31552426

[30] Younes M , Ostrowski M, Soiferman M, et al Odds ratio product of sleep EEG as a continuous measure of sleep state. Sleep. 2015;38(4):641–654. doi: https://doi.org/10.5665/sleep.4588 25348125 PMC4355904

[31] Berry RB , et al The AASM Manual for the Scoring of Sleep and Associated Events: Rule, Terminology and Technical Specifications, Version 2.2. Darien, IL: American Academy of Sleep Medicine; 2015.

[32] Mander BA , Winer JR, Walker MP. Sleep and human aging. Neuron. 2017;94(1):19–36. doi: https://doi.org/10.1016/j.neuron.2017.02.004 28384471 PMC5810920

[33] Beaudin AE , Raneri JK, Ayas NT, Skomro RP, Smith EE, Hanly PJ; Canadian Sleep and Circadian Network. Contribution of hypercapnia to cognitive impairment in severe sleep-disordered breathing. J Clin Sleep Med. 2022;18(1):245–254. doi: https://doi.org/10.5664/jcsm.9558 34286691 PMC8807902

[34] Bastien CH , Vallières A, Morin CM. Validation of the Insomnia Severity Index as an outcome measure for insomnia research. Sleep Med. 2001;2(4):297–307. doi: https://doi.org/10.1016/s1389-9457(00)00065-4 11438246

[35] The International Restless Legs Syndrome Study Group. Validation of the International Restless Legs Syndrome Study Group rating scale for restless legs syndrome. Sleep Med. 2003;4(2):121–132. doi: https://doi.org/10.1016/S1389-9457(02)00258-7 14592342

[36] Johns MWA. new method for measuring daytime sleepiness: the Epworth sleepiness scale. Sleep. 1991;14(6):540–545. doi: https://doi.org/10.1093/sleep/14.6.540 1798888

[37] Buysse DJ , Reynolds CF, Monk TH, Berman SR, Kupfer DJ. The Pittsburgh Sleep Quality Index: a new instrument for psychiatric practice and research. Psychiatry Res. 1989;28(2):193–213. doi: https://doi.org/10.1016/0165-1781(89)90047-4 2748771

[38] Nasreddine ZS , Phillips NA, Bédirian V, et al The montreal cognitive assessment, MoCA: a brief screening tool for mild cognitive impairment. J Am Geriatr Soc. 2005;53(4):695–699. doi: https://doi.org/10.1111/j.1532-5415.2005.53221.x 15817019

[39] Rey A. L’examen psychologique dans les cas d’encéphalopathie traumatique. (Les problems.). [The psychological examination in cases of traumatic encepholopathy. Problems.]. Arch Psychol. 1941;28:215–285.

[40] Wechsler D. Wechsler Adult Intelligence Scale – Fourth Edition. San Antonio, TX: Pearson; 2008.

[41] Malhotra A , Younes M, Kuna ST, et al Performance of an automated polysomnography scoring system versus computer-assisted manual scoring. Sleep. 2013;36(4):573–582. doi: https://doi.org/10.5665/sleep.2548 23565003 PMC3612255

[42] Younes M , Thompson W, Leslie C, Egan T, Giannouli E. Utility of technologist editing of polysomnography scoring performed by a validated automatic system. Ann Am Thorac Soc. 2015;12(8):1206–1218. doi: https://doi.org/10.1513/AnnalsATS.201411-512OC 26065574

[43] Goldschmied JR , Lacourse K, Maislin G, et al Spindles are highly heritable as identified by different spindle detectors. Sleep. 2021;44(4). doi: https://doi.org/10.1093/sleep/zsaa230 PMC8033448 33165618

[44] Younes M. The case for using digital EEG analysis in clinical sleep medicine. Sleep Sci Pract. 2017;1:2. doi: https://doi.org/10.1186/s41606-016-0005-0

[45] Wang D , Yee BJ, Wong KK, et al Comparing the effect of hypercapnia and hypoxia on the electroencephalogram during wakefulness. Clin Neurophysiol. 2015;126(1):103–109. doi: https://doi.org/10.1016/j.clinph.2014.04.012 24875233

[46] Younes M , Schweitzer PK, Griffin KS, Balshaw R, Walsh JK. Comparing two measures of sleep depth/intensity. Sleep. 2020;43(12). doi: https://doi.org/10.1093/sleep/zsaa127 32729619

[47] Allen RP , Picchietti D, Hening WA, Trenkwalder C, Walters AS, Montplaisi J; Restless Legs Syndrome Diagnosis and Epidemiology workshop at the National Institutes of Health. Restless legs syndrome: diagnostic criteria, special considerations, and epidemiology. Sleep Med. 2003;4(2):101–119. doi: https://doi.org/10.1016/s1389-9457(03)00010-8 14592341

[48] Grandner MA , Patel NP, Gehrman PR, Perlis ML, Pack AI. Problems associated with short sleep: bridging the gap between laboratory and epidemiological studies. Sleep Med Rev. 2010;14(4):239–247. doi: https://doi.org/10.1016/j.smrv.2009.08.001 19896872 PMC2888649

[49] Strauss E , et al A compendium of neuropsychological tests: Administration, norms, and commentary, 3rd ed. New York, NY: Oxford University Press; 2006.

[50] Gale SD , Baxter L, Connor DJ, Herring A, Comer J. Sex differences on the rey auditory verbal learning test and the brief visuospatial memory test-revised in the elderly: normative data in 172 participants. J Clin Exp Neuropsychol. 2007;29(5):561–567. doi: https://doi.org/10.1080/13803390600864760 17564921

[51] Smith EE , O'Donnell M, Dagenais G, et al; PURE Investigators. Early cerebral small vessel disease and brain volume, cognition, and gait. Ann Neurol. 2015;77(2):251–261. doi: https://doi.org/10.1002/ana.24320 25428654 PMC4338762

[52] Kane SP. Top 250 Drugs: Drug List by Therapeutic Category. ClinCalc LLC. https://clincalc.com/Downloads/Top250Drugs-DrugList.pdf Accessed November 27, 2019.

[53] Lusic Kalcina L , Pavlinac Dodig I, Pecotic R, Valic M, Dogas Z. Psychomotor performance in patients with obstructive sleep apnea syndrome. Nat Sci Sleep. 2020;12:183–195. doi: https://doi.org/10.2147/NSS.S234310 32210650 PMC7069561

[54] van Buuren S , et al Mice: multivariate imputation by chained equations in R. J Stat Soft. 2011;45(3):1–67. doi: https://doi.org/10.18637/jss.v045.i03

[55] Pedersen AB , Mikkelsen EM, Cronin-Fenton D, et al Missing data and multiple imputation in clinical epidemiological research. Clin Epidemiol. 2017;9:157–166. doi: https://doi.org/10.2147/CLEP.S129785 28352203 PMC5358992

[56] Hayes AF. Introduction to mediation, moderation, and conditional process analysis: a regression-based approach. 3rd ed. New York, NY: Methodology in the Social Sciences. The Guilford Press; 2022.

[57] Ho D , et al MatchIt: Nonparametric preprocessing for parametric causal inference. J Stat Soft. 2011;42(8):1–28. doi: https://doi.org/10.18637/jss.v042.i08

[58] Pishgar F , Greifer N, Leyrat C, Stuart E. MatchThem:: matching and weighting after multiple imputation. The R Journal. 2021;13(2):228–305. doi: https://doi.org/10.32614/rj-2021-073

[59] Baron RM , Kenny DA. The moderator-mediator variable distinction in social psychological research: conceptual, strategic, and statistical considerations. J Pers Soc Psychol. 1986;51(6):1173–1182. doi: https://doi.org/10.1037//0022-3514.51.6.1173 3806354

[60] Yu Q , Wu X, Li B, Scribner RA et al General multiple mediation analysis with an application to explore racial disparities in breast cancer survival. Journal Biomet Biostat. 2014;5(2):1000189. doi: https://doi.org/10.4172/2155-6180.1000189

[61] Abdullah H , Maddage NC, Cosic I, Cvetkovic D. Cross-correlation of EEG frequency bands and heart rate variability for sleep apnoea classification. Med Biol Eng Comput. 2010;48(12):1261–1269. doi: https://doi.org/10.1007/s11517-010-0696-9 21046273

[62] Himanen S-L , Virkkala J, Huupponen E, Hasan J. Spindle frequency remains slow in sleep apnea patientsthroughout the night. Sleep Med. 2003;4(4):361–366. doi: https://doi.org/10.1016/s1389-9457(03)00155-2 14592314

[63] Ondze B , Espa F, Dauvilliers Y, Billiard M, Besset A. Sleep architecture, slow wave activity and sleep spindles in mild sleep disordered breathing. Clin Neurophysiol. 2003;114(5):867–874. doi: https://doi.org/10.1016/s1388-2457(02)00389-9 12738432

[64] Purcell SM , Manoach DS, Demanuele C, et al Characterizing sleep spindles in 11,630 individuals from the National Sleep Research Resource. Nat Commun. 2017;8:15930. doi: https://doi.org/10.1038/ncomms15930 28649997 PMC5490197

[65] Parker JL , Melaku YA, D'Rozario AL, et al The association between obstructive sleep apnea and sleep spindles in middle-aged and older men: a community-based cohort study. Sleep. 2022;45(3). doi: https://doi.org/10.1093/sleep/zsab282 34850237

[66] Huupponen E , Himanen SL, Hasan J, Värri A. Automatic analysis of electro-encephalogram sleep spindle frequency throughout the night. Med Biol Eng Comput. 2003;41(6):727–732. doi: https://doi.org/10.1007/BF02349981 14686599

[67] Mohammadi H , Aarabi A, Rezaei M, Khazaie H, Brand S. Sleep spindle characteristics in obstructive sleep apnea syndrome (OSAS). Front Neurol. 2021;12:598632. doi: https://doi.org/10.3389/fneur.2021.598632 33716919 PMC7947924

[68] Chervin RD , Burns JW, Ruzicka DL. Electroencephalographic changes during respiratory cycles predict sleepiness in sleep apnea. Am J Respir Crit Care Med. 2005;171(6):652–658. doi: https://doi.org/10.1164/rccm.200408-1056OC 15591467

[69] .Xavier P, Behbehani K, Watenpaugh D, Burk JR. Detecting Electroencephalography Variations Due to Sleep Disordered Breathing Events. 2007 29th Annual International Conference of the IEEE Engineering in Medicine and Biology Society. Lyon, France, 2007; pp. 6097–6100. doi: https://doi.org/10.1109/IEMBS.2007.4353740 18003406

[70] Guilleminault C , Do Kim Y, Chowdhuri S, Horita M, Ohayon M, Kushida C. Sleep and daytime sleepiness in upper airway resistance syndrome compared to obstructive sleep apnoea syndrome. Eur Respir J. 2001;17(5):838–847. doi: https://doi.org/10.1183/09031936.01.17508380 11488314

[71] Fernandez LMJ , Lüthi A. Sleep spindles: mechanisms and functions. Physiol Rev. 2020;100(2):805–868. doi: https://doi.org/10.1152/physrev.00042.2018 31804897

[72] Chen C , Wang K, Belkacem AN, et al A comparative analysis of sleep spindle characteristics of sleep-disordered patients and normal subjects. Front Neurosci. 2023;17:1110320. doi: https://doi.org/10.3389/fnins.2023.1110320 37065923 PMC10098120

[73] Fogel SM , Smith CT. The function of the sleep spindle: a physiological index of intelligence and a mechanism for sleep-dependent memory consolidation. Neurosci Biobehav Rev. 2011;35(5):1154–1165. doi: https://doi.org/10.1016/j.neubiorev.2010.12.003 21167865

[74] Fang Z , Sergeeva V, Ray LB, Viczko J, Owen AM, Fogel SM. Sleep spindles and intellectual ability: epiphenomenon or directly related? J Cogn Neurosci. 2017;29(1):167–182. doi: https://doi.org/10.1162/jocn_a_01034 27626227

[75] Landry S , Anderson C, Andrewartha P, Sasse A, Conduit R. The impact of obstructive sleep apnea on motor skill acquisition and consolidation. J Clin Sleep Med. 2014;10(5):491–496. doi: https://doi.org/10.5664/jcsm.3692 24910549 PMC4046361

[76] Stevens D , Leong CWY, Cheung H, et al Sleep spindle activity correlates with implicit statistical learning consolidation in untreated obstructive sleep apnea patients. Sleep Med. 2021;86:126–134. doi: https://doi.org/10.1016/j.sleep.2021.01.035 33707093

[77] Barner C , et al Memory consolidation in fragmented sleep. Somnologie. 2016;20(1):37–46. doi: https://doi.org/10.1007/s11818-016-0041-0

[78] Yaffe K , Laffan AM, Harrison SL, et al Sleep-disordered breathing, hypoxia, and risk of mild cognitive impairment and dementia in older women. JAMA. 2011;306(6):613–619. doi: https://doi.org/10.1001/jama.2011.1115 21828324 PMC3600944

[79] Marchi NA , Solelhac G, Berger M, et al Obstructive sleep apnoea and 5-year cognitive decline in the elderly. Eur Respir J. 2023;61(4):2201621. doi: https://doi.org/10.1183/13993003.01621-2022 36796834 PMC10133583

[80] Chou KT , Chang Y-T, Chen Y-M, et al The minimum period of polysomnography required to confirm a diagnosis of severe obstructive sleep apnoea. Respirology. 2011;16(7):1096–1102. doi: https://doi.org/10.1111/j.1440-1843.2011.02022.x 21762445

[81] Baril A-A , Gagnon K, Brayet P, et al Obstructive sleep apnea during REM sleep and daytime cerebral functioning: a regional cerebral blood flow study using high-resolution SPECT. J Cereb Blood Flow Metab. 2020;40(6):1230–1241. doi: https://doi.org/10.1177/0271678X18814106 30465610 PMC7238367

[82] D’Rozario AL , Hoyos CM, Wong KKH et al Improvements in cognitive function and quantitative sleep EEG in OSA after Six Months of CPAP treatment. Sleep. 2022;45(6). doi: https://doi.org/10.1093/sleep/zsac013 PMC9189957 35029691

[83] Gratton MKP , Hamilton NA, Gerardy B, Younes M, Mazzotti DR. Wake intrusions in the electroencephalogram: a novel application of the odds ratio product in identifying subthreshold arousals. Sleep. 2024;47(5). doi: https://doi.org/10.1093/sleep/zsae039 PMC13032128 38334721

[84] Younes M , Hanly PJ. Immediate postarousal sleep dynamics: an important determinant of sleep stability in obstructive sleep apnea. J Appl Physiol. 2016;120(7):801–808. doi: https://doi.org/10.1152/japplphysiol.00880.2015 26718786

[85] Younes M , Gerardy B, Pack AI, Kuna ST, Castro-Diehl C, Redline S. Sleep architecture based on sleep depth and propensity: patterns in different demographics and sleep disorders and association with health outcomes. Sleep. 2022;45(6). doi: https://doi.org/10.1093/sleep/zsac059 PMC9195236 35272350

[86] Hayes AF. Beyond Baron and Kenny: statistical mediation analysis in the new millennium. Commun Monographs. 2009;76(4):408–420. doi: https://doi.org/10.1080/03637750903310360

[87] O’Rourke HP , MacKinnon DP. Reasons for testing mediation in the absence of an intervention effect: a research imperative in prevention and intervention research. J Stud Alcohol Drugs. 2018;79(2):171–181. doi: https://doi.org/10.15288/jsad.2018.79.171 29553343 PMC6019768

[88] Fairchild AJ , McDaniel HL. Best (but oft-forgotten) practices: mediation analysis. Am J Clin Nutr. 2017;105(6):1259–1271. doi: https://doi.org/10.3945/ajcn.117.152546 28446497 PMC5445681

[89] Johnson KA , Gordon CJ, Chapman JL, et al The association of insomnia disorder characterised by objective short sleep duration with hypertension, diabetes and body mass index: a systematic review and meta-analysis. Sleep Med Rev. 2021;59:101456. doi: https://doi.org/10.1016/j.smrv.2021.101456 33640704

[90] Alzheimer Society of Canada. Prevalence and Monetary Costs of Dementia in Canada: Population Health Expert Panel. Toronto, ON: Alzheimer Society of Canada; 2016.

[91] 2022 Alzheimer’s disease facts and figures. Alzheimers Dement. 2022;18(4):700–789. doi: https://doi.org/10.1002/alz.12638 35289055

