# Supplementary material for: Association between sleep microarchitecture and cognition in obstructive sleep apnea
Source: Sleep. 2024 Jun 29;47(12):zsae141. doi: 10.1093/sleep/zsae141 (PMC11632191; doi:10.1093/sleep/zsae141)
Supplement: zsae141_suppl_Supplementary_Tables_S1-S2_Figures_S1-S4 [file zsae141_suppl_supplementary_tables_s1-s2_figures_s1-s4.pdf]

ONLINE SUPPLEMENT

Association between sleep microarchitecture and cognition in obstructive sleep apnea

Andrew E. Beaudin[1,2], Magdy Younes[3,4], Bethany Gerardy[4], Jill K. Raneri[5], AJ Marcus Hirsch Allen[6], Teresa Gomes[7], Simon Gakwaya[8], Fréderic Series[8], John Kimoff[7], Robert P. Skomro[9], Najib T. Ayas[6], Eric E. Smith[1,2] and Patrick J Hanly[2,5,10] on behalf of the Canadian Sleep and Circadian Network (CSCN)

[1] Department of Clinical Neurosciences, Cumming School of Medicine, University of Calgary, Calgary, AB, Canada
[2] Hotchkiss Brain Institute, Cumming School of Medicine, University of Calgary, Calgary, AB, Canada
[3] Sleep Disorders Center, Misericordia Health Center, University of Manitoba, Winnipeg, Canada.
[4] YRT Limited, Winnipeg, Manitoba, Canada.
[5] Sleep Centre, Foothills Medical Centre, Calgary AB, Canada
[6] Department of Medicine, Respiratory and Critical Care Divisions, University of British Columbia, Vancouver, BC, Canada
[7] Respiratory Division and Sleep Laboratory, McGill University Health Centre, Montreal, QC, Canada
[8] Unité de recherche en pneumologie, Centre de recherche, Institut Universitaire de Cardiologie et de Pneumologie de Québec, Université Laval, Québec, QC, Canada
[9] Division of Respirology, Critical Care and Sleep Medicine, University of Saskatchewan, Saskatoon, SK, Canada
[10] Department of Medicine, Cumming School of Medicine, University of Calgary, Calgary, AB, Canada

To Whom Correspondence Should Be Addressed:

Patrick J. Hanly, MD, FRCPC, D,ABSM
Professor, Department of Medicine
Sleep Centre, Foothills Medical Centre
Cumming School of Medicine
University of Calgary
Health Sciences Centre, Rm 1421
3330 Hospital Drive NW
Calgary, Alberta, Canada T2N 4N1
Tel: +1 403-210-8743
Fax +1 403-283-6151
Email: phanly@ucalgary.ca

Supplemental Methods

Study Protocol

The following academic sleep centers participated: The Sleep Centre at Foothills Medical Centre (University of Calgary, Calgary, Alberta), the Sleep Disorders Centre at Saskatoon City Hospital (University of Saskatchewan, Saskatoon, Saskatchewan) and the University of British Columbia Hospital Sleep Disorders Clinic (Vancouver, British Columbia), the McGill University Health Centre Sleep Disorders Centre (McGill University, Montreal, Quebec), and the Sleep Laboratory at Institut Universitaire de Cardiologie et de Pneumologie de Québec (Université Laval, Laval Quebec).

At the University of Calgary, McGill University and Université Laval, OSA was diagnosed by either unattended home sleep apnea testing (HSAT) or in-laboratory polysomnography (PSG). Participants completed a single daytime study visit consisting of a sleep questionnaire and cognitive testing prior to any treatment for OSA.

At the University of Saskatchewan, OSA was diagnosed by in-lab PSG. Participants were recruited at the sleep laboratory on the morning following their PSG and completed a sleep questionnaire and cognitive testing the same morning.

At the University of British Columbia, OSA was diagnosed by in-lab PSG. Participants were recruited on the evening they came to the sleep laboratory and completed a sleep questionnaire and cognitive testing prior to their PSG.

Sleep questionnaire

The sleep questionnaire included questions related to age, sex, height, weight, ethnicity, lifestyle (cigarette smoking, caffeine and alcohol consumption), medical history, comorbidities

(self-reported history of physician diagnosed hypertension, coronary artery disease, heart failure, stroke, high cholesterol, diabetes, atrial fibrillation and kidney disease), medications, family history of medical disease, sleep schedule, sleep related symptoms (e.g., snoring, witnessed apneas) and co-exiting restless legs syndrome (RLS) and insomnia. Daytime sleepiness and sleep quality were assessed with the Epworth Sleepiness Scale (ESS) [1]] and the Pittsburgh Sleep Quality Index (PSQI [2]), respectively.

Presence or absence of insomnia was determined using the Insomnia Severity Index (ISI [3]), which consists of 5 questions related to the severity of sleep-onset and sleep maintenance difficulties, satisfaction with current sleep patterns, the degree to which sleep problems interfere with daily functioning, the noticeability of sleep problems to others and the degree of worry or distress a patient has about their sleep problems. The total score ranges from 0 to 28 with a score of 0-7 indicating no clinical insomnia; 8-14 indicating sub-threshold insomnia; 15-21 indicating moderate insomnia; and 22-28 indicating severe insomnia. Therefore, participants were categorized as having insomnia if they scored ≥15.

Patients were categorized as having RLS if they reported having all of the following symptoms [4,5]: an urge to move their legs due to uncomfortable or unpleasant sensations in their legs that begin or worsen during periods of rest or sleep and are improved by movement, and that are worse in the evening or night, or only occur during the evening and night. RLS severity was quantified using an ordinal scale (range: 1-7) indicating the number of times the above symptoms occur: 1 = less than one time per year, 2 = at least one time a year, but less than one time per month, 3 = one time per month, 4 = 2-4 times per month, 5 = 2-3 times per week, 6 = 4-5 times per week and 7 = 6-7 times per week [4].

Cognitive testing

Global cognitive function was assessed with the Montreal Cognitive Assessment (MoCA) [6], while episodic memory was assessed using the Rey Auditory Verbal Learning Test [7] (RAVLT) and information processing speed was measured with the Wechsler Adult Intelligence Scale-4th Edition Digit–Symbol Coding (DSC) subtest [8].

The MoCA is a screening tool for cognitive impairment that assesses multiple cognitive domains including visuospatial/executive function, semantic knowledge, memory, attention and concentration, language, abstract and conceptual thinking, and orientation. It takes ~10 minutes to administer and has a maximum score of 30. An adjustment for low education is incorporated into the final score by adding one point to a participant's score if they have ≤12 years of formal education, up to a maximum of 30; a score <26 indicates mild cognitive impairment (MCI) [6].

The RAVLT is a list-learning paradigm and takes ~30-35 minutes to administer including a 20-minute break. Briefly, the examiner reads aloud a list of 15 words (List A) to the participant, who is asked to immediately recall as many of the words as possible (in any order), reciting them back to the examiner for scoring. The examiner reads List A for five consecutive trials with the participant recalling as many words as possible immediately after the examiner completes the list. For the 6th trial, the examiner reads aloud a list of 15 different, unrelated words to those in List A (List B) after which the participant is asked to immediately recall as many words from List B as possible. Following the List B trial, the participant is asked to recall as many words from List A as possible. Next, a 20-minute break is provided after which the participant is asked to recall as many words from List A as possible. The total number of words correctly recalled is the delayed free recall score.

The DSC subtest is a paper-and-pencil test that evaluates information processing speed, but also involves attention, short-term memory, concentration, learning, psychomotor speed, visuoperception and motor speed [8]. At the top of a piece of paper, a key is provided which pairs the numbers 1-9 with a unique symbol. Below the key are several rows of numbers. Using the key, the participant draws the matching symbol in an empty space below each number. A participant's final score is the total number of correctly matched number-symbol pairs completed in two minutes.

References

1. Johns MW. A new method for measuring daytime sleepiness: the Epworth sleepiness scale. *Sleep*. 1991;**14**(6):540-545. doi:10.1093/sleep/14.6.540
2. Buysse DJ*, et al*. The Pittsburgh Sleep Quality Index: a new instrument for psychiatric practice and research. *Psychiatry Res*. 1989;**28**(2):193-213. doi:10.1016/0165-1781(89)90047-4
3. Bastien CH*, et al*. Validation of the Insomnia Severity Index as an outcome measure for insomnia research. *Sleep Med Clin*. 2001;**2**(4):297-307. doi:10.1016/S1389-9457(00)00065-4
4. Allen RP*, et al*. Restless legs syndrome: diagnostic criteria, special considerations, and epidemiology. *Sleep Med*. 2003;**4**(2):101-119. doi:10.1016/S1389-9457(03)00010-8
5. The International Restless Legs Syndrome Study Group. Validation of the International Restless Legs Syndrome Study Group rating scale for restless legs syndrome. *Sleep Med*. 2003;**4**(2):121-132. doi:10.1016/S1389-9457(02)00258-7
6. Nasreddine ZS*, et al*. The Montreal Cognitive Assessment, MoCA: A Brief Screening Tool For Mild Cognitive Impairment. *J Am Geriatr Soc*. 2005;**53**(4):695-699. doi:10.1111/j.1532-5415.2005.53221.x
7. Rey A. L'examen psychologique dans les cas d'encéphalopathie traumatique. (Les problems.). [The psychological examination in cases of traumatic encepholopathy. Problems.]. *Arch Psychol*. 1941;**28**:215-285.
8. Wechsler D. *Wechsler Adult Intelligence Scale – Fourth Edition*. Pearson; 2008

Supplemental Results

Table S1 Cognitive scores for the entire cohort and categorized by OSA severity.

	**Entire Cohort**	**No/Mild OSA**	**Moderate OSA**	**Severe OSA**
**MoCA**				
N	1,142	443	257	442
Total Score	25.6 ± 3.0	26.0 ± 2.9	25.7 ± 3.0	25.1 ± 3.0[*†]
Total < 26, n (%)	494 (43)	164 (37)	103 (40)	227 (51)[*]
Component Scores				
Visuospatial/Executive	4.4 ± 0.8	4.4 ± 0.9	4.3 ± 0.9	4.4 ± 0.8
Naming	2.9 ± 0.3	2.9 ± 0.3	2.9 ± 0.4	2.9 ± 0.4
Attention	5.5 ± 0.9	5.5 ± 0.9	5.5 ± 0.9	5.5 ± 0.8
Language	2.3 ± 0.8	2.3 ± 0.9	2.5 ± 0.7	2.3 ± 0.9[*†]
Abstraction	1.6 ± 0.6	1.7 ± 0.6	1.7 ± 0.6	1.5 ± 0.7[*†]
Delayed Recall	2.7 ± 1.7	3.1 ± 1.6	2.7 ± 1.7[*]	2.2 ± 1.6[*†]
Orientation	5.9 ± 0.3	5.9 ± 0.4	5.9 ± 0.3	5.9 ± 0.3
**RAVLT**				
N	1,082	411	242	429
Delayed Recall (z-score)	-0.3 ± 1.1[a]	-0.2 ± 1.1	-0.3 ± 1.2[a]	-0.3 ± 1.1[a]
**WAIS-IV**				
N	1,101	419	246	436
DSC (z-score)	-0.6 ± 0.9[a]	-0.5 ± 1.0[a]	-0.6 ± 1.0[a]	-0.7 ± 0.9[a*]

Mean ± SD; [*]p≤0.05 vs No OSA; [†]p≤0.05 vs Mild OSA; [¶]p≤0.05 vs Moderate OSA; [a]p≤0.05 vs zero.
Abbreviations: MoCA, Montreal Cognitive Assessment; RAVLT, Rey Auditory Verbal Learning Test; WAIS-IV, Weschler Adult Intelligence Scale 4th Edition; DSC, digit symbol coding.

Table S2 Standardized parameter estimates (β (95% CI)), unadjusted p-value and false discovery adjusted p-value (q-value) for linear associations between MoCA total, RAVLT delayed recall and DSC scores with overall normalized EEG power (EEG_{NP}) and the Delta:Alpha ratio (D/A ratio) adjusted for age, sex, low education (except MoCA), total sleep time and OSA severity group in primary and secondary analysis cohorts.

Dependent Variable	MoCA Total Score	P-value	Q-value	RAVLT Delayed Recall	P-value	Q-value	DSC	P-value	Q-value
Primary Analyses									
N	*1142*			*1082*			*1101*		
EEG_{NP}	0.06 (-0.001 – 0.12)	0.055	0.166	0.01 (-0.05 – 0.07)	0.688	0.855	0.02 (-0.04 – 0.08)	0.568	0.855
D/A Ratio	-0.005 (-0.06 – 0.05)	0.855	0.855	0.06 (0.005 – 0.12)	0.033	0.166	-0.009 (-0.07 – 0.05)	0.770	0.855
Secondary Analyses									
N	*619*			*575*			*582*		
EEG_{NP}	0.07 (-0.01 – 0.15)	0.094	0.282	-0.01 (-0.09 – 0.07)	0.789	0.946	0.02 (-0.06 – 0.10)	0.628	0.942
D/A Ratio	-0.03 (-0.11 – 0.05)	0.486	0.942	0.10 (0.02 – 0.19)	0.015	0.088	-0.001 (-0.09 – 0.09)	0.987	0.987

Abbreviations: MoCA, Montreal Cognitive Assessment; RAVLT, Rey Auditory Verbal Learning Test; DSC, Weschler Adult Intelligence Scale 4th Edition Digit Symbol Coding.

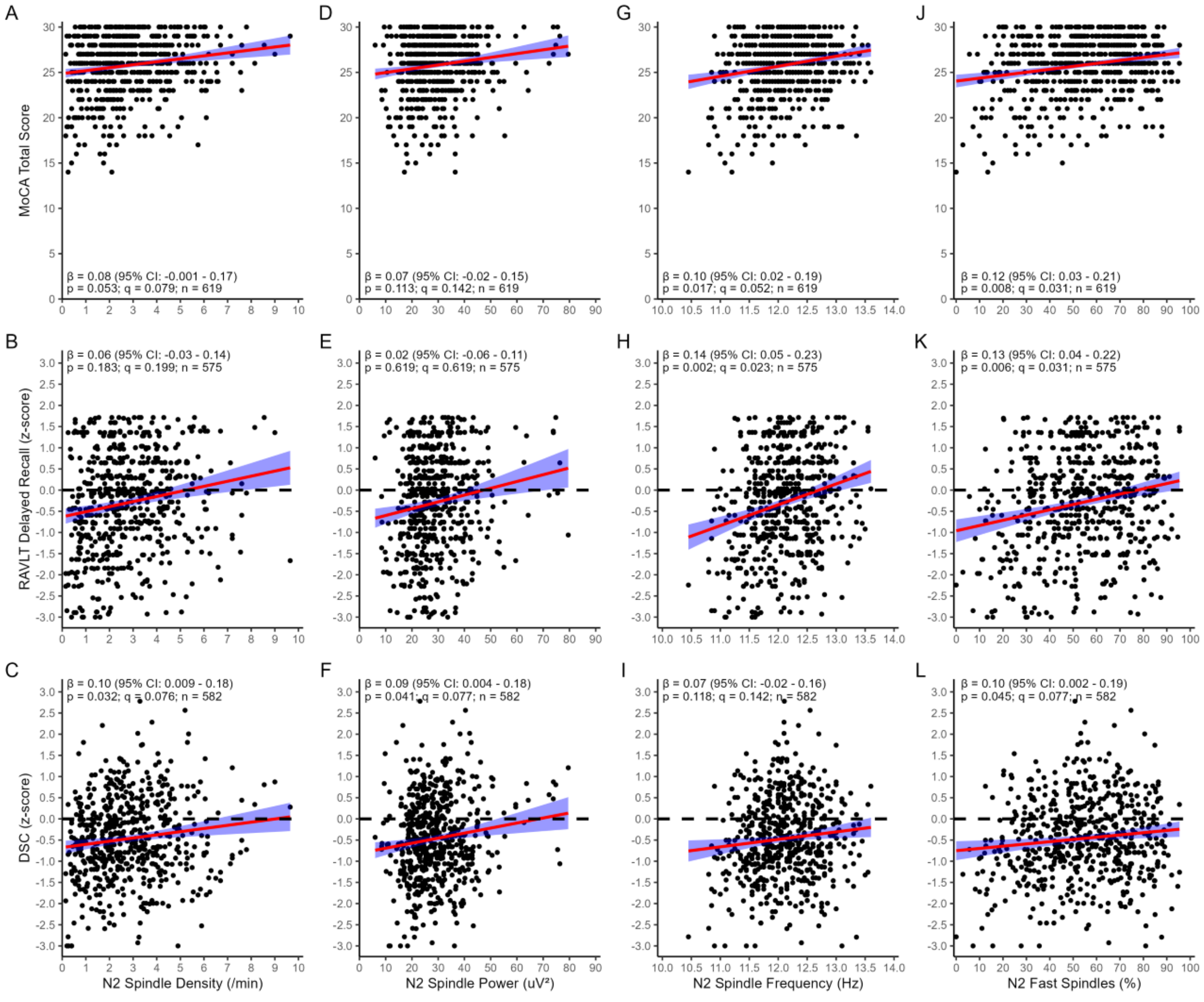

Figure S1 Linear associations between cognitive scores and N2 spindle density (A-C), power (D-F), frequency (G-I), and percentage of fast spindles (J-L) in our <u>*secondary analysis cohort*</u> of participants who underwent full-night polysomnography. Standardized parameter estimates (β) and 95% CI provided in plots reflect the relationships between cognitive scores and spindle characteristics adjusting for age, sex, education (except MoCA), total sleep time and OSA severity group.

Abbreviations: DSC, Weschler Adult Intelligence Scale 4th Edition WAIS-IV digit symbol coding; MoCA, Montreal cognitive assessment; RAVLT, Rey auditory verbal learning.

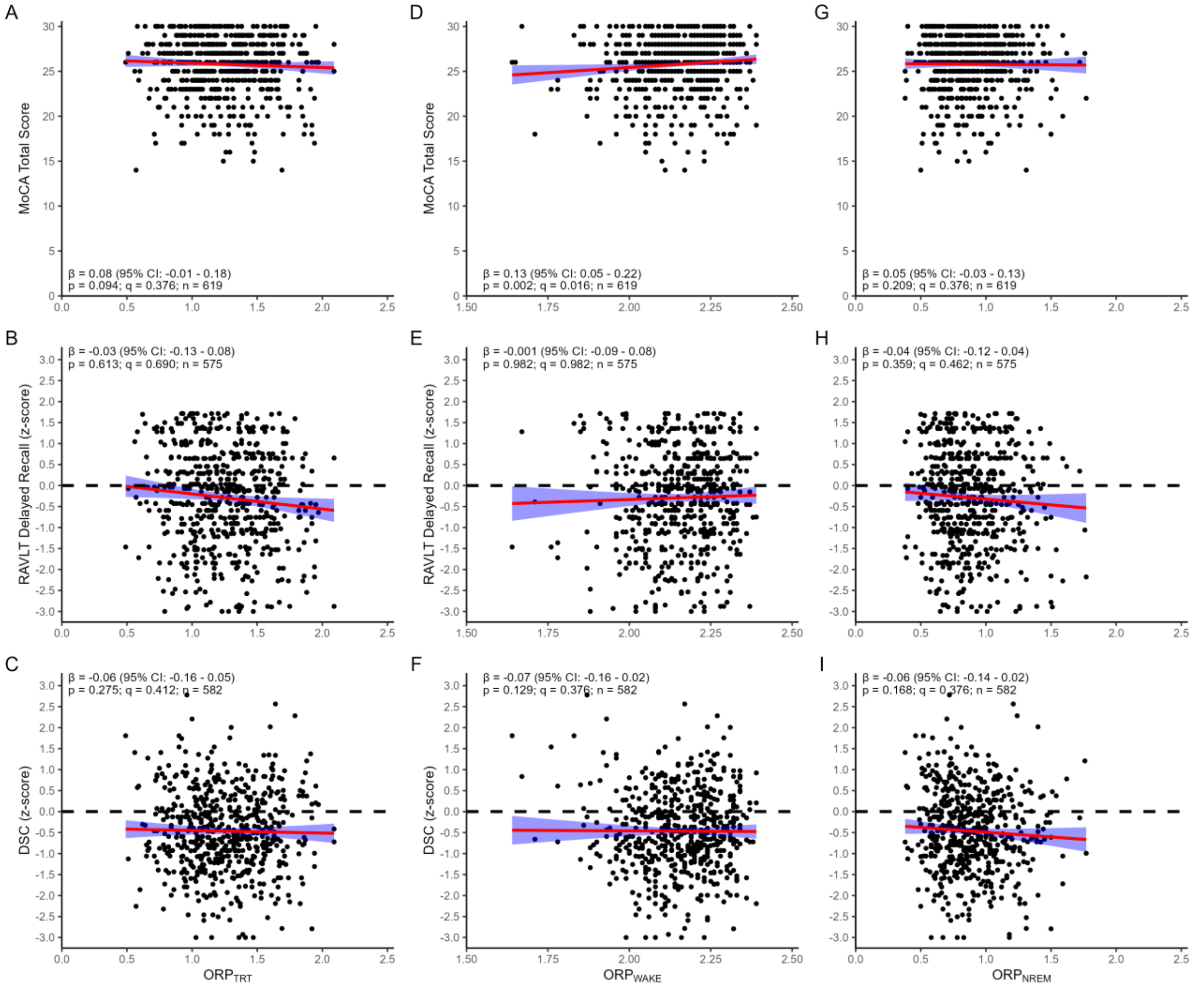

Figure S2 Linear associations between cognitive scores and the odds ratio product for total recording time (ORP_{TRT}; A-C), during wakefulness (ORP_{WAKE}; D-F) and during non-rapid eye movement sleep (ORP_{NREM}; G-I) in our *secondary analysis cohort* of participants who underwent full-night polysomnography. Standardized parameter estimates (β) and 95% CI provided in plots reflect the relationships between cognitive scores and ORP adjusting for age, sex, education (except MoCA), total sleep time and OSA severity group.

Abbreviations: DSC, Weschler Adult Intelligence Scale 4th Edition WAIS-IV digit symbol coding; MoCA, Montreal cognitive assessment; RAVLT, Rey auditory verbal learning

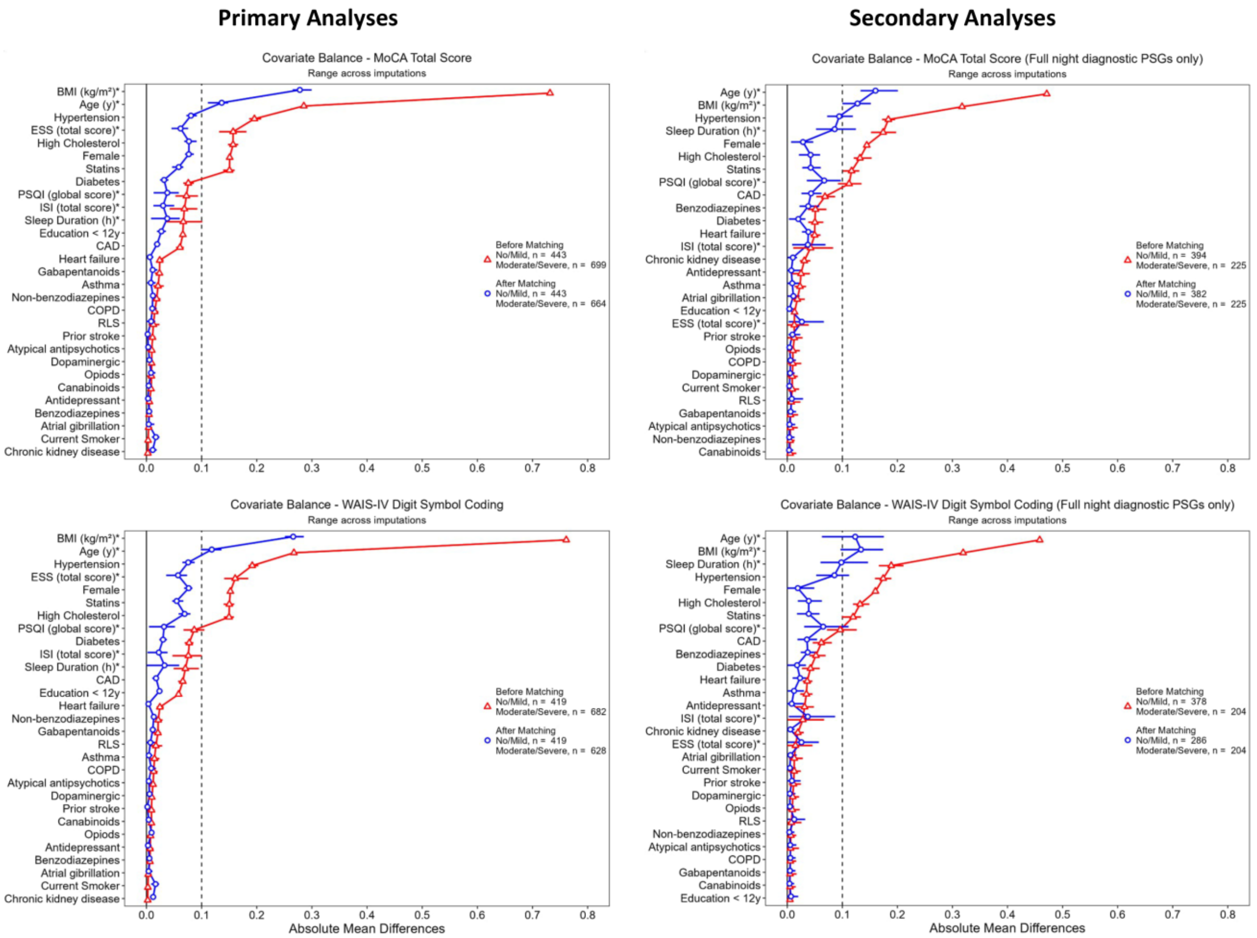

Figure S3 Covariate balance between the no/mild and moderate/severe OSA groups before (red triangles) and after (blue circles) propensity score matching across all 50 imputed data sets in our primary and secondary analyses for participants with a Montreal cognitive assessment (MoCA) total score (top panel) and Weschler Adult Intelligence Scale 4th Edition (WAIS-IV) digit symbol coding score (bottom panel) prior to mediation analyses. Covariates were considered adequately matched if the mean difference was ≤0.1 (dashed vertical line). Asterik (*) indicates continuous variables where absolute mean difference is reported as the standardized mean difference.

Abbreviations: BMI, body mass index; CAD, coronary artery disease; CKD, chronic kidney disease; COPD, chronic obstructive pulmonary disease; ESS, Epworth sleepiness scale; ISI, insomnia severity index; PSQI, Pittsburgh sleep quality index; RLS, restless leg syndrome.

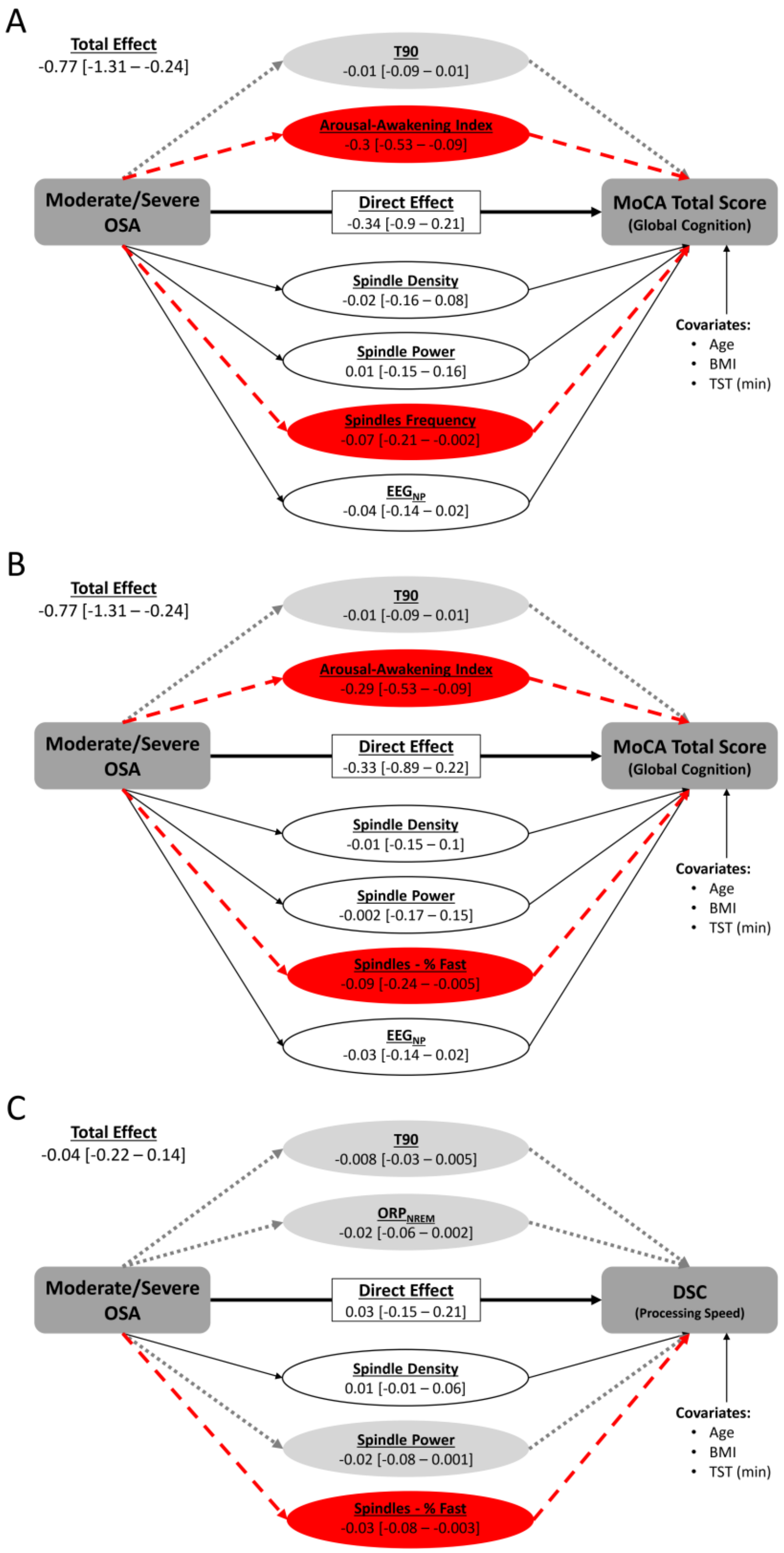

Figure S4 Mediation analyses assessing the effect of moderate/severe OSA on the Montreal cognitive assessment (MoCA) total score (A, spindle frequency included in the mediation model; B, spindles %-fast included in the mediation model) and the Weschler Adult Intelligence Scale 4th Edition WAIS-IV digit symbol coding (DSC) score (C) in our *secondary analysis cohort* of participants who underwent full-night polysomnography. Values are the mean effects and 95% bias-corrected and accelerated confidence intervals pooled across all 50 imputed data sets (10,000 bootstrap iterations per data set).

Abbreviations: % Fast, percentage of spindles with a frequency of 12-16 Hz; BMI, body mass index; EEG$_{NP}$, overall normalized EEG power; ORP$_{NREM}$, odds-ratio product during non-rapid eye movement sleep; T90, percentage of total sleep time with oxyhemoglobin saturation <90%; and TST, total sleep time.
